# Addressing TB multimorbidity in policy and practice: An exploratory survey of TB providers in 27 high-TB burden countries

**DOI:** 10.1371/journal.pgph.0001205

**Published:** 2022-12-07

**Authors:** Alexander Jarde, Noemia Siqueira, Saima Afaq, Farah Naz, Muhammad Irfan, Pervaiz Tufail, Faiza Aslam, Olamide Todowede, Shagoofa Rakhshanda, Humaira Khalid, Yan Lin, Olivia Bierman, Asma Elsony, Helen Elsey, Najma Siddiqi, Kamran Siddiqi

**Affiliations:** 1 Department of Health Sciences, University of York, York, United Kingdom; 2 Institute of Public Health and Social Sciences, Khyber Medical University, Peshawar, Pakistan; 3 Department of Epidemiology and Biostatistics, School of Public Health, Imperial College London, London, United Kingdom; 4 Centre for Injury Prevention and Research Bangladesh (CIPRB), Dhaka, Bangladesh; 5 Department of Mental Health, Psychiatry & Behavioural Sciences, Peshawar Medical College, Peshawar, Pakistan; 6 National group of TB people, TB and Poverty Subgroup Core Team, Stoptb.org., Pakistan; 7 Institute of Psychiatry, Rawalpindi Medical University, Rawalpindi, Pakistan; 8 International Union Against Tuberculosis and Lung Disease, Paris, France; 9 Department of Global Public Health, Karolinska Institute, Solna, Sweden; 10 Epi-Lab: The Epidemiological Laboratory, Khartoum, Sudan; 11 Hull York Medical School, University of York, York, United Kingdom; 12 Bradford District Care NHS Foundation Trust, Shipley, United Kingdom; University of Melbourne Department of Public Health: The University of Melbourne School of Population and Global Health, AUSTRALIA

## Abstract

In people with TB, co-existence of long-term conditions (e.g., depression, diabetes and HIV) and risk factors (e.g.,alcohol misuse, malnutrition, and smoking) are associated with increased mortality and poor treatment outcomes including delayed recovery, TB treatment failure and relapse. However, it is unclear as to what extent these comorbidities are addressed in TB policy and practice. Between August and October 2021, we conducted an online cross-sectional survey in high-TB burden countries. We recruited a purposive sample of TB health workers, managers, policy makers, advisors and advocates from these countries. The survey enquired about the extent to which various comorbid conditions are: (a) mentioned in TB policies, plans, and guidelines; (b) screened, diagnosed, treated or referred to specialist services by TB healthcare workers. We summarised using descriptive analysis. Of the 1100 potential respondents contacted in 33 countries, 543 responded but only 446 (41%) from 27 countries provided sufficient data for inclusion in the study. We found no notable differences between these providing insufficient data and those completing the survey. HIV, diabetes mellitus, depression and tobacco and alcohol use disorders were identified as the most common and concerning comorbid conditions in TB. HIV was screened for and managed by TB services in most countries. Screening for diabetes and/or tobacco and alcohol use disorders was offered by almost half of all TB services but only a few offered relevant treatments. Depression was rarely screened for, almost never treated, and only infrequently referred to specialist services. Most respondents felt confident in screening/diagnosing these comorbid conditions but not in treating these conditions. With the exception of HIV, chronic comorbid conditions are only partially screened for and rarely managed within TB services. Mental health conditions are for the most part neglected. Given their adverse impact on TB outcomes, integrating screening and management of these comorbidities within TB programmes offers a significant opportunity to meet TB targets, address non-communicable diseases and improve patient well-being.

## Introduction

In 2020, tuberculosis (TB) caused more deaths worldwide (1.5 million) than any other infectious disease [1]. A range of other long-term conditions (e.g., depression, diabetes and HIV) and risk factors (e.g., alcohol misuse, malnutrition, and smoking) that commonly co-occur with TB are associated with increased mortality amongst the TB patients and these conditions and risk factors may also worsen its clinical course, delay recovery and lead to TB treatment failure and relapse [2]. TB multimorbidity, defined as the co-occurrence of TB and one or more chronic conditions in a single individual at one point in time, is also a growing global concern [2]. There is some (albeit limited) information available on the patterns and risks of multimorbidity compared to the general population, and on the burden of disease associated with TB multimorbidity. According to an analysis of the World Health Survey in 48 low- and middle-Income countries (LMIC), the prevalence of one or more non-communicable diseases was almost twice as high among those with TB than those without (68.8% vs. 34.4%) [3]. A number of meta-analyses have estimated the prevalence of various comorbidities; about half of TB patients in LMIC have depression [4], about one third in Sub-Saharan Africa have HIV [5], and worldwide, around 15% have diabetes [6]. Furthermore, TB multimorbidity is associated with increased symptom complexity, healthcare use and costs, and significantly worse treatment outcomes (odds ratio of 1.98 [95%CI, 1.56–2.52]), including increased mortality (relative risk upto 4 [95%CI, 3.3–4.6]), compared with TB alone [3, 7–11]. An analysis of the world health survey in 48 LMICs showed that at least one third of the the YLDs in those who had TB multimorbidity were attributable to non communicable diseases [3]. There is a growing need to address TB multimorbidity, and to offer patient-centred, integrated care for multiple conditions [12, 13]. However, it is unclear to what extent current TB services identify and manage these other mental and physical comorbidities in patients with presumptive and/or confirmed TB. While the WHO and STOP TB partnership recognises the significant impact of comorbid conditions on TB outcomes [14], the extent to which these are detected and managed is not reported even for high TB-burden countries. Our aim was to understand whether TB multimorbidity is currently considered and addressed in high-TB burden countries [15]. Our key objectives were to, overall: 1) understand the extent to which TB multimorbidity was considered in policy, management and services within TB healthcare; 2) assess which specific comorbidities were being considered; and 3) identify potential gaps in service provision for TB multimorbidity.

## Methods

We conducted a cross-sectional multi-country online survey between August and October 2021. This study was approved by the Research Governance Committee of the University of York.

### Inclusion and exclusion criteria

The survey was conducted in 33 countries; 30 were classified as high-TB burden countries by the WHO for 2021–2025, *while* the other three (Cambodia, Russia and Zimbabwe) were part of the previous list but removed in this update [15]. Our key informants were TB health workers, managers, policy makers, advisors and employees of TB advocacy organisations in these countries. For TB health workers, we included people based in primary, secondary and tertiary care TB services, in either the public or the private (for profit or not for profit) sector, in a high-TB burden country. Respondents could be in a management, clinical or advisory/advocacy role. In addition, TB programme managers, supervisors and coordinators at the national, provincial, regional and district level were also included.

### Sampling strategy

Given the exploratory nature of this study, a formal sample size was not estimated; instead, we aimed to recruit as many respondents as possible from as many countries as possible in the time period available for data collection (August-October 2021).

We used a variety of strategies to identify the widest range of potential respondents including using targeted emails to people identified through online searching e.g., Stop TB Partnership Partners’ Directory (http://www.stoptb.org/partners/). We asked members of the Tuberculosis Multimorbidity Network (https://www.impactsouthasia.com/tbmm/), representatives of The Union and the World Health Organization, researchers in the field and TB programme managers for potential key informants. We also included social platforms (Twitter) to publicise the survey widely.

We recruited a purposive sample of key respondents in management, advisory, and clinical positions at different levels of the public and private systems offering TB services in each high-burden country.

### Informed consent

An Informed consent was obtained from all participants.

### Ethics statement

Ethical Approval was obtained from Health Sciences Research Governance Committee, Department of Health Sciences, University of York (YO10 5DD), United Kingdom having approval number: ***HSRGC/2021/458/A***.

### Data collection

We invited potential respondents to participate in the survey via email. In addition to the link to the survey, the invite included information about the survey, rights of the participants and arrangements for data confidentiality and security. Engaging with the survey was taken as consent for participation. A reminder email was sent halfway through the data collection period.

The online survey (developed using Qualtrics) included the following sections: (1) respondent’s characteristics, including their country, organisation and role (but no personal information such as age, gender, or socioeconomic status); (2) relevant policies, strategic plans, and clinical guidelines in their country (e.g., whether such documents existed, what comorbidities were mentioned in them and what were clinicians asked to do); (3) how comorbid conditions were considered in respective TB services/clinics (e.g., which comorbidities were diagnosed, screened for, and treatment offered); and (4) what were the three most common and concerning comorbidities in their experience and how capable they felt in diagnosing or treating them (see S1 Appendix- ‘TBMM survey preview’ in the online supplementary materials). The survey was available in English, French and Portuguese.

### Data analysis

We excluded any incomplete surveys where no responses were provided for sections 2 to 4 (*i*.*e*., only characteristics of the respondent were provided). We summarised quantitative and qualitative responses using descriptive analyses. Given the heterogeneity in the number and type of respondents for each country, the non-random, non-representative sampling procedure, and the small sample size, we decided against pooling responses from all countries together. The main analysis only included countries with ≥10 responses to avoid findings being skewed due to too few responses. Results including all countries are provided in S2 Appendix—‘TBMM survey all country results’ in online supplementary materials [1].

For items referring to policy documents and clinical guidelines relevant to wider geographical regions (provinces or countries), we made the following assumption: If >50% of respondents of a country stated that policy documents and clinical guidelines in that country contained certain information (e.g., requirement of clinicians to screen for a certain comorbidity), we projected this to the whole country.

In order to facilitate the interpretation of the results, we collapsed certain items together: Responses to diagnosis and screening questions were collated in a single item, diagnosis/screening; a positive response to either one of these two items was considered positive for the new item. The same strategy was used to combine items asking about the start and maintenance of care (start/maintain care) and items asking about referral to, or liaison with a specialised service for a comorbidity (referral/liaison).

Further details can be found in the online supplement (Report).

## Results

**Fig 1:**
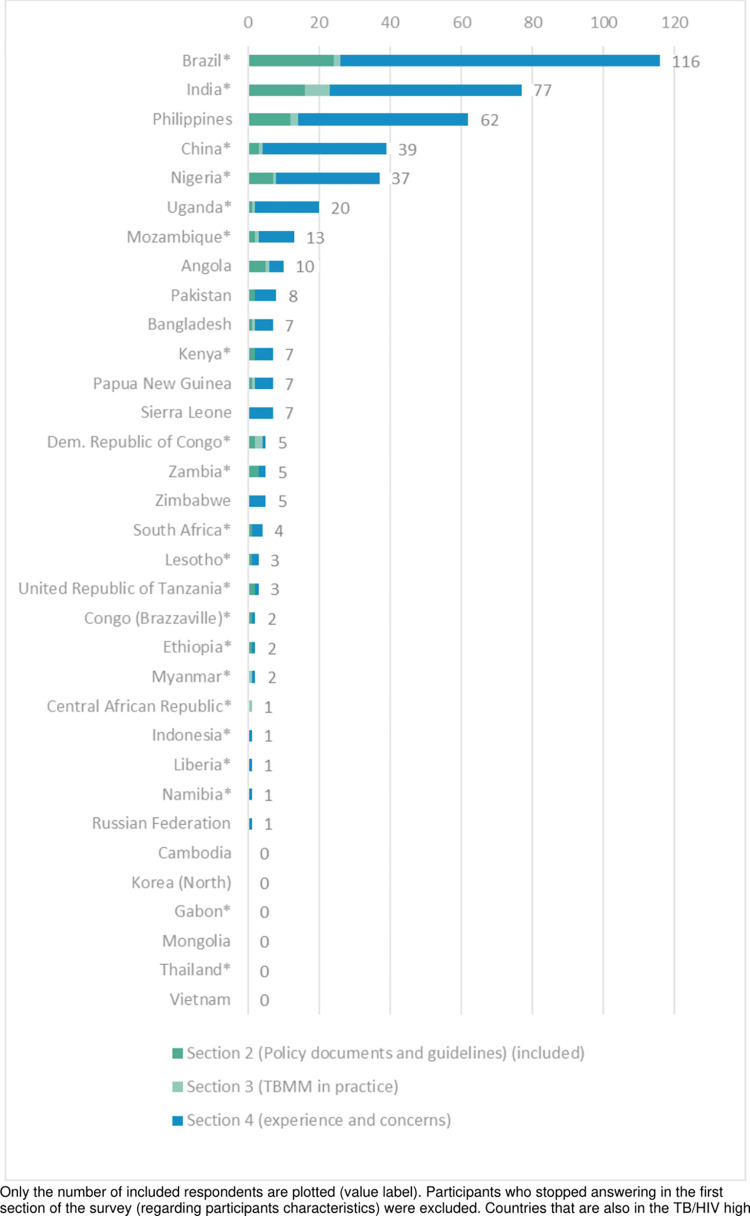
Number of respondents completing sections of the survey by country. Only the number of included respondents are plotted (value label). Participants who stopped answering in the first section of the survey (regarding participants characteristics) were excluded. Countries that are also in the TB/HIV high burden list are indicated with an asterisk [*].

We invited 1,100 prospective respondents to participate in the survey. 543 responded, of whom 18% (97) did not engage beyond section 1 (respondent’s characteristics) and their data were excluded. A further 16.4% (89) and 3.7% (20) did not answer any questions beyond sections 2 and 3, respectively; this left 62.1% (337) respondents who completed the full survey (Fig 1). When we compared the characteristics of excluded and included respondents, we found no notable differences (S3 Appendix—’respondents’ and non-respondents’ characteristics’ in the online supplementary materials).

### Respondents’ characteristics

We obtained responses from 27 countries out of the 33 countries, with two-thirds of all respondents from four countries (Brazil: 116, India: 77, the Philippines: 62, and China: 39) (Table 1). We obtained more than 10 responses from three other countries (Nigeria: 37, Uganda: 20 and Mozambique: 13), whilst the remaining 20 countries (Angola, Pakistan, Bangladesh, Kenya, Papua New Guinea, Sierra Leone, Democratic Republic of Congo, Zambia, Zimbabwe, South Africa, Lesotho, United Republic of Tanzania, Congo-Brazzaville, Ethiopia, Myanmar, Central African Republic, Indonesia, Liberia, Namibia, Russian Federation) offered 10 or fewer responses; no response was received from six countries (Cambodia, Democratic People’s Republic of Korea, Gabon, Mongolia, Thailand, Vietnam) (Fig 1).

**Table 1:** Characteristics of respondents.

Country	frequency	%
Brazil	90	26.7
India	54	16.0
Philippines	48	14.2
China	35	10. 4
Nigeria	29	8.6
Uganda	18	5.3
Other	27	18.7
**Position**		
TBP manager	158	35.4
Consultant	61	13.7
1y.health care	71	15.9
2y.health care	25	5.6
3y.health care	64	14.3
Other	67	15.0
**Do you work at a DOTS clinic? (health care workers only)**		
Yes	83	51.9
No	71	44.4
.	6	3.6
**Main role in the organisation**		
Service manager	105	24.0%
Clinician/ TB healthc	162	36.0%
Advocacy / advisory	78	17.0%
Other (please specify	93	21.0%
NR	8	2.0%
**Type of service provider**		
public	117	52.0%
private (not-for-prof	28	12.0%
private (for-profit)	7	3.0%
Other	5	2.0%
NR	70	31.0%
**At what level are you working? (TBP managers and Consultants only**)		
International	17	5.8
National	64	21.8
Provincial	43	14.7
State	56	19.1
District	77	26.3
Subdistrict	36	12.3
**Work in a TB clinic?**		
Yes, I do work in a TB clinic	182	52.2
No, I do not work in a TB clinic	167	47.8

**Fig 2:**
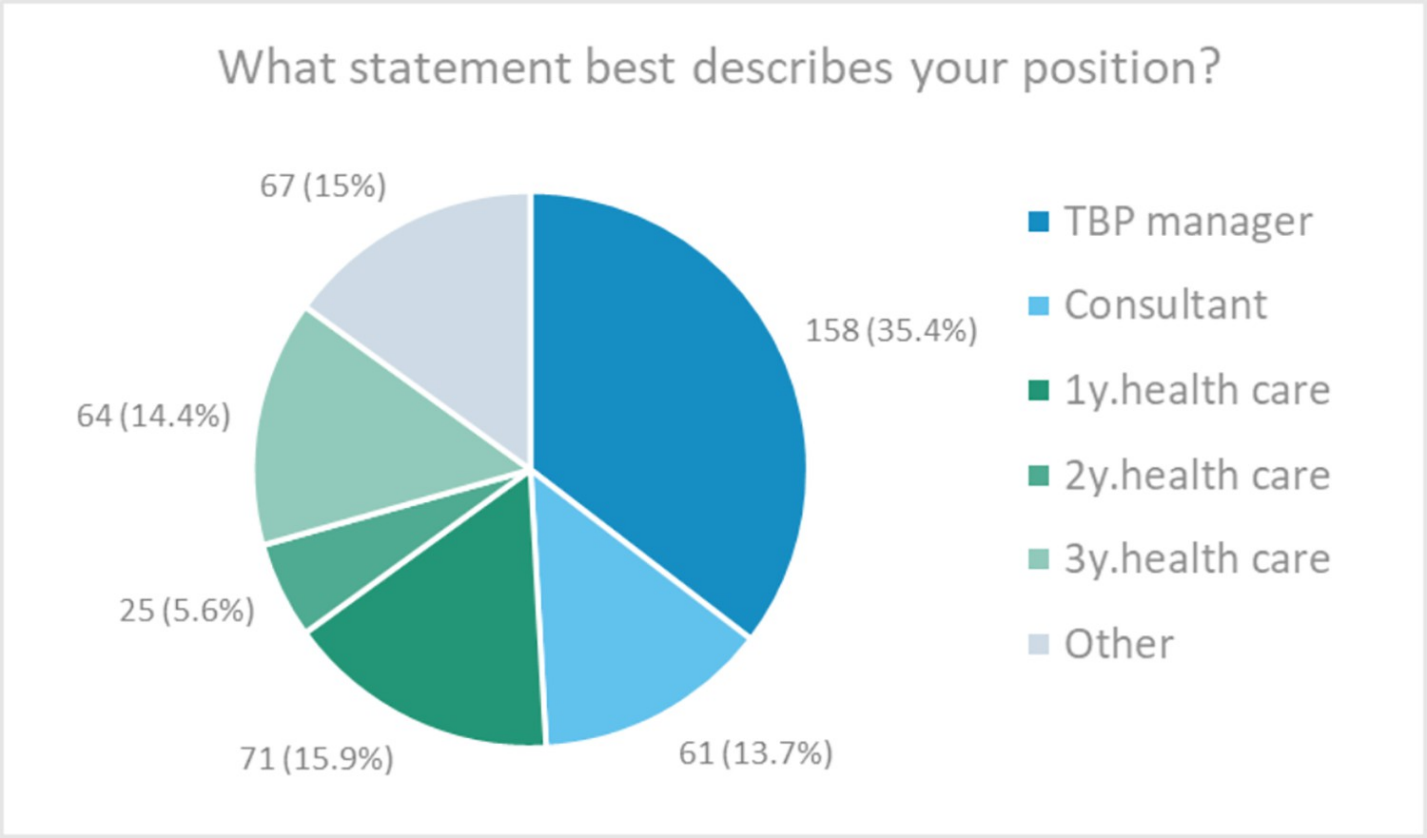
Positions of respondents.

Thirty-five percent of respondents described themselves as ‘TB programme manager/ supervisor/ coordinator’ (referred henceforth as *National TB Programme [NTP] managers*), and a similar proportion (36%) described themselves as working directly with people with TB in primary, secondary, or tertiary health care. Half of the remaining respondents (14%) were ‘the UNION/ WHO/ other non-governmental organisation consultants/ advocates/ advisors’ referred henceforth as *Consultant*s (Fig 2). When asked specifically about their role in the organisation or TB service, 162 (36%) described it as ‘clinician/ TB healthcare professional’, 105 (24%) as ‘service manager’ and 78 (17%) as ‘advocacy or advisory’ (Table 1). This indicates that not all health care workers had a clinical role, and not all NTP managers described their role as a service manager (Table C in S3 Appendix—’respondents and non-respondents’ characteristics’ in the online supplementary materials).

**Fig 3:**
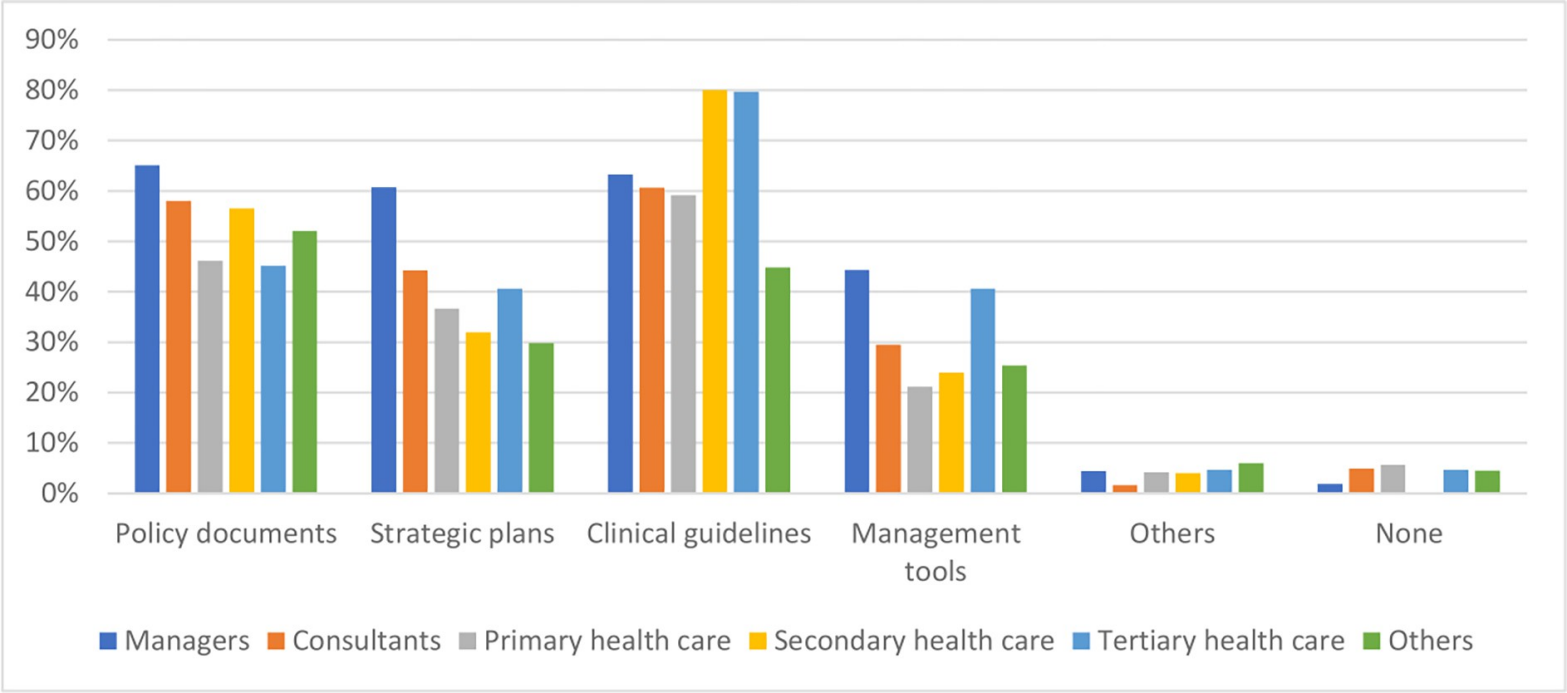
Proportion of TB health professionals who thought that multimorbidity is mentioned in TB policies and guidelines.

HIV, diabetes mellitus, depression, tobacco use, and alcohol misuse disorders were consistently mentioned as the most common and concerning comorbid conditions or risk factors in people with TB. Most TB health professionals recalled multimorbidity mentioned in TB clinical guidelines and a slightly fewer proportion thought it was mentioned in TB policies (Fig 3). TB programme managers were more likely to refer to policy documents and strategic plans as opposed to clinicians who mostly remembered it being mentioned in clinical guidelines.

### HIV

Most (22) TB high-burden countries included in our survey were also listed under TB/HIV high-burden countries. In countries with >10 responses, survey respondents reported that HIV was mentioned in TB policy documents and clinical guidelines (Table 2); and that in practice, HIV was screened for in people with TB. A high proportion of respondents in these countries also stated that care for HIV was started or maintained, and TB services referred to, or liaised with specialist HIV services, or did both (Table 3). Most of the respondents felt capable of diagnosing HIV and slightly fewer felt capable of treating it (Table 4). The above pattern was repeated across all countries included in the survey with most respondents recalling integration of HIV diagnosis and management within TB policy and practice (Tables 2–4).

**Table 2:** Proportion of countries where majority (>50%) thought that specific comorbidities are included within TB policy documents.

Comorbidities*	Countries	Prevention	Screening	Treatment
**HIV**	Brazil	70%	84%	79%
	Philippines	66%	96%	62%
	India	61%	64%	62%
	China	69%	64%	83%
	Nigeria	87%	94%	94%
	Uganda	89%	84%	89%
	Mozambique	91%	91%	91%
	All 27 countries	74%	85%	85%
**Diabetes Mellitus**	Brazil	56%	69%	67%
	Philippines	57%	98%	74%
	India	38%	51%	49%
	China	64%	61%	78%
	Nigeria	13%	19%	26%
	Uganda	42%	68%	58%
	Mozambique	55%	64%	73%
	All 27 countries	33%	56%	48%
**Depression**	Brazil	25%	31%	25%
	Philippines	43%	55%	42%
	India	38%	36%	41%
	China	50%	36%	56%
	Nigeria	10%	6%	6%
	Uganda	42%	37%	21%
	Mozambique	55%	82%	73%
	All 27 countries	11%	19%	15%
**Tobacco**	Brazil	54%	62%	53%
	Philippines	57%	72%	32%
	India	61%	46%	33%
	China	56%	44%	42%
	Nigeria	13%	10%	10%
	Uganda	58%	32%	21%
	Mozambique	55%	27%	0%
	All 27 countries	44%	22%	7%
**Alcohol**	Brazil	51%	53%	44%
	Philippines	57%	62%	30%
	India	59%	43%	36%
	China	53%	39%	44%
	Nigeria	19%	16%	16%
	Uganda	63%	37%	26%
	Mozambique	55%	27%	0%
	All 27 countries	41%	22%	4%

*Only countries with ≥10 responses and only the five most common and concerning conditions are presented (see Supplement for all countries and all conditions)

**Table 3:** Proportion of countries where majority (>50%) thought that specific comorbidities are managed within TB services.

Comorbidities*	Countries**	Diagnose/Screen	Start/Maintain/Care	Refer/liaise
**HIV**	Brazil	87%	59%	80%
	Philippines	86%	62%	84%
	India	62%	54%	43%
	China	86%	64%	78%
	Nigeria	97%	83%	83%
	Uganda	84%	74%	53%
	Mozambique	91%	82%	82%
	All 27 countries	89%	74%	70%
**Diabetes Mellitus**	Brazil	71%	59%	45%
	Philippines	80%	72%	58%
	India	52%	43%	33%
	China	86%	83%	56%
	Nigeria	13%	3%	27%
	Uganda	68%	32%	47%
	Mozambique	36%	18%	73%
	All 27 countries	52%	26%	48%
**Depression**	Brazil	43%	32%	52%
	Philippines	40%	26%	60%
	India	31%	26%	26%
	China	44%	44%	61%
	Nigeria	10%	3%	20%
	Uganda	26%	32%	47%
	Mozambique	55%	18%	73%
	All 27 countries	19%	7%	30%
**Tobacco**	Brazil	66%	45%	51%
	Philippines	58%	22%	30%
	India	52%	30%	28%
	China	53%	53%	31%
	Nigeria	13%	3%	3%
	Uganda	53%	26%	32%
	Mozambique	55%	18%	45%
	All 27 countries	48%	11%	15%
**Alcohol**	Brazil	68%	37%	52%
	Philippines	52%	22%	28%
	India	49%	26%	28%
	China	50%	56%	33%
	Nigeria	13%	3%	3%
	Uganda	42%	11%	26%
	Mozambique	55%	18%	64%
	All 27 countries	30%	7%	19%

*Only countries with ≥10 responses and only the five most common and concerning conditions are presented (see Supplement for all countries and all conditions).

**Responses: Brazil = 92; India = 61; Philippines = 50; China = 36; Nigeria = 30; Uganda = 19; Mozambique = 11

**Table 4:** Proportion of countries where majority (>50%) of TB health professionals thought that they are capable of managing specific comorbidities.

Comorbidities*	Countries**	Capable of diagnosing	Capable of treating
**HIV**	Brazil	94%	40%
	Philippines	93%	10%
	India	81%	62%
	China	84%	45%
	All 19 countries	79%	63%
**Diabetes Mellitus**	Brazil	79%	53%
	Philippines	93%	79%
	India	54%	54%
	China	94%	68%
	All 19 countries	74%	68%
**Depression**	Brazil	49%	13%
	Philippines	62%	31%
	India	50%	46%
	China	48%	23%
	All 19 countries	26%	5%
**Tobacco**	Brazil	81%	45%
	Philippines	62%	34%
	India	46%	46%
	China	65%	42%
	All 19 countries	47%	16%
**Alcohol**	Brazil	74%	15%
	Philippines	55%	21%
	India	46%	46%
	China	68%	52%
	All 19 countries	47%	16%

*Only countries with ≥10 responses and only the five most common and concerning conditions are presented (see *S2* Appendix for all countries and all conditions)

**Responses: Brazil = 47; China = 31; India = 26; Philippines = 29

### Diabetes mellitus

In countries with >10 responses, diabetes was mentioned in TB policy documents and clinical guidelines except for Nigeria (Table 2). In practice, most TB service providers reported diagnosing/screening for diabetes in people with TB but fewer reported starting/maintaining care or referring to or liaising with specialist diabetes services. Only in Mozambique, most respondents reported people with TB and diabetes were referred to specialist services (Table 3). While most respondents felt capable of diagnosing diabetes in people with TB, again, fewer felt capable of treating this condition (Table 4).

When considering all countries with at least one response, diabetes was considered in practice in about half of the countries (Table 3). A slightly higher proportion of respondents felt capable of diagnosing and treating TB (Table 3).

### Depression

In countries with >10 responses, depression was rarely mentioned in TB policy documents and clinical guidelines (with the exception of Mozambique where clinicians were asked to prevent, screen and treat depression in TB services) (Table 2). Consequently, in practice, depression was neither screened for nor diagnosed in any of these countries according to most respondents. In Mozambique, little over half of the respondents (55%) reported screening or diagnosis of depression in TB services. Likewise, most respondents reported that depression was not treated in TB services in any of these countries. However, in Brazil, China, Mozambique and Philippines, most patients were at least referred to or offered liaison with mental health services. While approximately half of the respondents (service providers) felt capable of diagnosing depression, most were not confident in treating depression (Table 4). The above picture did not change by including all high-TB burden countries with >1 response in the sensitivity analysis (Tables 2–4) except that the vast majority of responding service providers felt incapable of diagnosing or treating depression.

### Tobacco use

Tobacco was mentioned in TB policy and clinical guidelines in all countries with 10 responses except Nigeria. However, prevention of tobacco, amongst those diagnosed with TB, use was the focus in most of these guidelines, with less attention on screening and cessation advice (Table 2). This was reflected in practice, with only about half of the respondents in these countries (fewer in Nigeria) reporting that patients were routinely asked about tobacco use. Even fewer reported that cessation advice/treatment or referral to cessation specialists was offered (Table 3). Only in four countries with 10 responses, respondents reported that they felt capable of asking about tobacco use; and less than half felt capable of treating tobacco addiction in people with TB (Table 4).

In only half of the countries with at least one response, people with TB were asked or advised about tobacco use and only in a few countries were patients treated for tobacco addiction or referred to specialist services (Tables 2–4). Similarly, only in about half of the countries a majority of respondents felt capable of screening for tobacco use, and in very few countries (3) did respondents feel capable of treating tobacco addiction (Tables 2–4).

### Alcohol use

The response pattern for alcohol use was very similar to that for tobacco use. Except for Nigeria, in all countries with 10 responses, clinicians were encouraged in policy documents and clinical guidelines, to diagnose and screen for alcohol use in TB patients. They reported less often that these documents included requirements to start/maintain care or to involve other specialist services for alcohol misuse (Table 2). In practice, alcohol use was diagnosed/screened for in about half of the services, with considerably fewer starting/maintaining care or referring/liaising with other specialist services (Table 3). Regarding how capable respondents felt about diagnosing alcohol use disorder in people with TB, the percentage ranged from 46% to 81% in the countries with 10 responses. However, most respondents did not feel capable of treating alcohol use disorder (Table 4).

In countries with at least one response, most clinicians reported screening for alcohol in TB patients, but people from only a small number of countries were treated (2 countries) or referred (5 countries) for alcohol use (Tables 2–4). In about one half of the countries, most respondents felt capable of screening for alcohol use disorder in TB patients. (Tables 2–4

## Discussion

The survey highlighted that HIV, diabetes, tobacco use, alcohol use disorders, and depression are the five most common and concerning comorbidities in people with TB. While the prevention, screening and treatment of HIV was considered within TB policies and guidelines across most countries, tobacco, alcohol, diabetes and depression were mentioned less often. At the level of service provision, most of the high-TB burden countries screened, diagnosed and offered referrals for HIV in people with TB. About half of the participating countries offered similar services or some levels of liaison with specialist services for comorbid diabetes. On the other hand, screening for tobacco and alcohol use disorders were conducted only in a minority of countries; very few countries initiated, maintained care or liaised with specialist services. Depression was consistently absent from policies and service provision in almost all countries.

The impact of HIV on TB, and its implications for TB and HIV control, have long been recognised as a global public health challenge. This is reflected in the level of integration of care for HIV in TB services observed in our survey. The WHO TB/HIV policy [13], published over a decade ago, was aimed at establishing and strengthening collaboration between HIV and TB control programmes. The goal was to reduce the burden of HIV in TB patients by providing HIV prevention, diagnosis and treatment through integrated TB and HIV services. In line with this policy, most high-TB/HIV burden countries have put policies and guidelines in place for the screening and management of HIV in TB patients. Our findings are in line with other literature highlighting that the TB-HIV collaborative care has been integrated at various levels; these range from referral-based approaches to a more integrated person-centred care model where TB-HIV services are provided under one roof [16, 17].

The WHO, in 2011, declared TB-diabetes collaborative care as one of its TB eradication strategies and included it as an essential part of the efforts to achieve Sustainable Development Goals (SDGs) [18]. Our findings suggest that most health care systems are yet to fully integrate diabetes care within TB care; more countries need to get involved in collaborative TB-diabetes management. Other researchers have also observed varying levels of collaborative care: for example, In Pakistan, the uptake of diabetes testing among TB cases was reported as 21.8% while the uptake of TB testing among individuals with pre-diabetes and diabetes has been reported to be as low as 4.7% [19]. This lack of TB-DM co-management or integrated care may be due to a lack of programmatic leadership, investment, training and workforce in diabetes care. However, other studies [20, 21] reveal that given adequate capacity-building programmes, identification and mitigation of operational challenges and provision of logistic supplies, there is good potential to increase awareness about TB-diabetes collaborative care, and provide TB-diabetes bi-directional screening, management and reporting.

Despite being recognised in the Diagnostic and Statistical Manual of Mental Disorders (DSM-5-TR) [22] tobacco and alcohol use disorders are rarely considered for further advice or treatment by clinicians offering TB care, as shown in our survey. Tobacco and alcohol use are important determinants of unfavourable TB treatment outcomes [23]. In 2007, WHO and the International Union Against TB and Lung Diseases, highlighted the dangerous interaction between TB and tobacco smoking and emphasised that tobacco control activities needs implementing as an integral part of TB management [24]. This interaction between TB and tobacco epidemics may account for over 15–20% of TB-related deaths [25]. Findings of the ASSIST trial [26], show that offering cessation support intervention is highly effective when delivered by TB DOTS (Directly Observed Treatment Short-course) facilitators. The role of TB DOTS facilitators in delivery of smoking cessation support effectively in a TB clinic has been acknowledged by other studies also [27]. and attempts to scale up tobacco cessation with national TB programmes are gaining ground [28]. Similarly, the relationship between TB and alcohol use disorder has been firmly established [29, 30]. Imtiaz et al estimated that in 2014 about 170 000 deaths due to TB were attributable to alcohol consumption worldwide, and as many as 17% of incident cases of TB and 15% of deaths due to TB could be prevented by eliminating the harmful use of alcohol [31]. Given the advantages of offering tobacco and alcohol cessation support to TB patients, approaches should be implemented for systematic screening and early identification and effective management, including referral to specialists for support.

Our findings suggest a lack of integrated depression services within the TB programme; these findings are in line with a recent (2019) global survey of national TB programme directors by Sweetland et al [32]. This semi-structured survey of national TB programme directors from 26 countries of all income levels reported that only two out of the twenty six national TB programmes included screening for any mental disorder. Similar to our findings, the survey reported that national TB programmes currently do not address mental disorders as part of routine practice. Nevertheless, receptivity was high, creating an opportunity to integrate the management of depression and other mental health problems within TB programmes.

### Strengths and limitations

Our sampling strategy resulted in over 400 responses from 27 of 33 high-burden countries but most responses (two-thirds) came from only four countries (Brazil, India, Philippines and China). Therefore, our findings are most relevant to these four countries and cannot be generalised across all high-TB burden countries. Furthermore, a lack of probability sampling meant that even the responses from each individual country may not necessarily be representative of that country’s policy and practice. In addition, 85% of the respondents represent government or UN agencies, which limits the possibility of triangulation between governmental and non-governmental sources. Since the survey was anonymous, we were unable to track who did and who did not answer our invitation and can therefore not assess the characteristics of non-respondents. However, we obtained responses from a wide range of TB programme managers, primary, secondary and tertiary health care workers, and consultants. We, therefore, believe that our results provide valuable initial insight into this topic, particularly for the countries with multiple respondents.

Despite the aforementioned limitations, this survey provides a useful snapshot of how conditions comorbid with TB are prioritised and addressed both in policy and guidelines, and in practice in high-TB-burden countries. We found little evidence of routine screening and reporting of comorbid conditions by TB services. Our findings highlight an important surveillance gap in the existing TB recording and reporting system. Although TB-HIV collaborative services have been embedded at various levels of the TB programme, there is a lack of non-communicable disease (NCD) integration within TB programmes (and limited evidence on evidence-based integrated care). Integrating NCD services within TB programmes may offer a viable solution to reduce TB and NCD-related disease burden. Global organisations, such as the WHO and The Union could help educate policy makers and programme managers through international conferences and through the dissemination of educational materials. Furthermore, the integration of NCD services into TB programmes also requires strong capacity-building efforts among primary care workers. An initial step for the TB programmes across the globe may be to provide and scale up routine screening of various NCDs. We also recommend development and evaluation of TB specific interventions targeting common comorbidities to help fill the knowledge gap on how to achieve integrated care and its effectiveness for people (with TB) with comorbidities.

## Supporting information

S1 Appendix TBMM survey qualtrics preview. (PDF)

S2 Appendix TBMM survey all country results. (XLSX)

S3 Appendix Characteristics of respondents and non-respondents. (PDF)

S1 File TBMM survey full report. (PDF)
