# Correction: Addressing TB multimorbidity in policy and practice: An exploratory survey of TB providers in 27 high-TB burden countries

**DOI:** 10.1371/journal.pgph.0002186

**Published:** 2023-07-12

**Authors:** Alexander Jarde, Noemia Siqueira, Saima Afaq, Farah Naz, Muhammad Irfan, Pervaiz Tufail, Faiza Aslam, Olamide Todowede, Shagoofa Rakhshanda, Humaira Khalid, Yan Lin, Olivia Biermann, Asma Elsony, Helen Elsey, Najma Siddiqi, Kamran Siddiqi

The twelfth author’s name is spelled incorrectly. The correct name is: Olivia Biermann.
