# Supplementary material for: Addressing TB multimorbidity in policy and practice: An exploratory survey of TB providers in 27 high-TB burden countries
Source: PLOS Glob Public Health. 2022 Dec 7;2(12):e0001205. doi: 10.1371/journal.pgph.0001205 (PMC10022227; doi:10.1371/journal.pgph.0001205)
Supplement: S1 Appendix — (PDF) [file pgph.0001205.s001.pdf]

Tools Saved at 11:49 AM Published Preview Publish

TB Multimorbidity Survey

iQ Score: Fair

Welcome and introduction

Q1

Tuberculosis Multimorbidity Survey

Thank you for your interest in participating in this study on tuberculosis multimorbidity (TB Multimorbidity), the co-occurrence of one or more chronic conditions in a single individual with tuberculosis.
The purpose of this study is to understand if any type of TB Multimorbidity is considered, either at the management level (national TB programme, clinical guidelines, etc.) or in the clinical practice (TB service providers). If any TB Multimorbidity is considered, we are also interested in knowing what conditions are considered and how they are taken into account.
We are interested in how TB Multimorbidity is considered at multiple levels of the system, which is why we are requesting participation from a variety of professionals in the health care system in each of the 30 high-TB burden countries (see the full list here). We would like to reach as many TB experts as possible in each country, including national or regional TB programme managers or coordinators, and clinicians working in TB service providers in primary, secondary, or tertiary health care services in both the public and the private sector (e.g. NGOs).

You can read more information about the study here.

If you are interested in participating in this survey, which only takes 5-10 minutes, please indicate your country and position below and click the '>>' button.

Q2 *

1- What country do you work in?

Angola

Q3

2- What state / province do you work in?

Q4

3- What statement **best** describes your position?

- I'm a TB programme manager/ supervisor/ coordinator
- I'm a UNION/ WHO/ other NGO's consultant/ advocate/ advisor for this country
- I work with people with TB in primary health care
- I work with people with TB in secondary health care
- I work with people with TB in tertiary health care
- Other (please specify):

Q30

Display this question

If 3- What statement best describes your position? I work with people with TB in primary health care Is Selected

Or 3- What statement best describes your position? I work with people with TB in secondary health care Is Selected

Or 3- What statement best describes your position? I work with people with TB in tertiary health care Is Selected

In Page Display Logic may behave unexpectedly with Validation Options

3b- Do you work at a DOTS clinic?

- Yes
- No

Import from library | Add new question

Add Block

Default Question Block

Q5

4- What is the name of the organisation / TB service provider you are involved in?

Q6

5- What is your main role in the organisation / TB service?

- Service manager
- Clinician/ TB healthcare professional
- Advocacy / advisory
- Other (please specify)

Q7

Display this question

If 3- What statement best describes your position? I work with people with TB in primary health care Is Selected

Or 3- What statement best describes your position? I work with people with TB in secondary health care Is Selected

Or 3- What statement best describes your position? I work with people with TB in tertiary health care Is Selected

6a- What type of TB service provider do you work at?

- a provider in the public sector
- a not-for-profit provider in the private sector
- a for-profit provider in the private sector
- * Other (please specify)

Q8

Display this question

If 3- What statement best describes your position? I'm a TB programme manager/ supervisor/ coordinator Is Selected

Or 3- What statement best describes your position? I'm a UNION/ WHO/ other NGO's consultant/ advocate/ advisor for this country Is Selected

6b- At what level are you working? (Check all that apply)

- International
- National
- Provincial
- State
- District
- Sub-district

Q9

Page 2 of 5

Import from library

Add new question

Add Block

Policies, strategic plans, and clinical guidelines.

Q10

For this survey we are defining tuberculosis multimorbidity as as the co-occurrence of one or more chronic conditions in a single individual with tuberculosis at one point in time. Given the variety in the definitions of the term ‘chronic’, we will use an inclusive definition as “something that is continuing or occurring again and again for a long time”.

In our research, common chronic conditions in people with TB include, but are not limited to, HIV, diabetes mellitus, hepatitis (B and C), depression and other mental health conditions, among others.
However, we are aware that this is not an exhaustive list, so *please don’t feel constrained by it and feel free to add the ones that are relevant in your context in the ‘other’ fields for each question*.

Q11

7- In your country, are any TB multimorbidities mentioned in any of the following documents?

- ☐ Policy documents
- ☐ Strategic plans
- ☐ Clinical guidelines
- ☐ Coordination management tools
- ☐ * Other (please specify): ______
- ☐ TB multimorbidities are not mentioned in any of these documents
- ☐ ⦸ Don't know

Q12

Display this question

If 7- In your country, are any TB multimorbidities mentioned in any of the following documents? Policy documents Is Selected

Or 7- In your country, are any TB multimorbidities mentioned in any of the following documents? Strategic plans Is Selected

Or 7- In your country, are any TB multimorbidities mentioned in any of the following documents? Clinical guidelines Is Selected

Or 7- In your country, are any TB multimorbidities mentioned in any of the following documents? Coordination management tools Is Selected

Or 7- In your country, are any TB multimorbidities mentioned in any of the following documents? Other (please specify): Is Selected

8- What conditions are mentioned, and what are clinicians asked to do?

	Prevention	Screening	Treatment	Other	Not addressed
HIV	☐	☐	☐	☐	☐
Diabetes Mellitus	☐	☐	☐	☐	☐
Hepatitis C virus	☐	☐	☐	☐	☐
Hepatitis B virus	☐	☐	☐	☐	☐
Depression	☐	☐	☐	☐	☐
Anxiety	☐	☐	☐	☐	☐
Tobacco use	☐	☐	☐	☐	☐
Alcohol use	☐	☐	☐	☐	☐
Chronic renal failure	☐	☐	☐	☐	☐
* Other (1): ____	☐	☐	☐	☐	☐
* Other (2): ____	☐	☐	☐	☐	☐
* Other (3): ____	☐	☐	☐	☐	☐

Q13

Display this question

If 7- In your country, are any TB multimorbidities mentioned in any of the following documents? Policy documents Is Selected

Or 7- In your country, are any TB multimorbidities mentioned in any of the following documents? Strategic plans Is Selected

Or 7- In your country, are any TB multimorbidities mentioned in any of the following documents? Clinical guidelines Is Selected

Or 7- In your country, are any TB multimorbidities mentioned in any of the following documents? Coordination management tools Is Selected

Or 7- In your country, are any TB multimorbidities mentioned in any of the following documents? Other (please specify): Is Selected

8b- Please feel free to add any additional details

____

Q14

Page 3 of 5

Import from library

Add new question

Add Block

Related to services and practice: Screening, diagnosis, surveillance, treatment

Q17

Display this question

If 7- In your country, are any TB multimorbidities mentioned in any of the following documents? Policy documents Is Selected

Or 7- In your country, are any TB multimorbidities mentioned in any of the following documents? Strategic plans Is Selected

Or 7- In your country, are any TB multimorbidities mentioned in any of the following documents? Clinical guidelines Is Selected

Or 7- In your country, are any TB multimorbidities mentioned in any of the following documents? Coordination management tools Is Selected

Or 7- In your country, are any TB multimorbidities mentioned in any of the following documents? Other (please specify): Is Selected

In a previous question we asked you about how TB Multimorbidity was taken into account in ${q://QID9/ChoiceGroup/SelectedChoices}, etc. However, in clinical practice the reality is often different from what is established in these documents. Therefore, in the following questions **we want to know how TB Multimorbidity is considered *in practice***.

Q18

Display this question

If 7- In your country, are any TB multimorbidities mentioned in any of the following documents? Policy documents Is Not Selected

And 7- In your country, are any TB multimorbidities mentioned in any of the following documents? Strategic plans Is Not Selected

And 7- In your country, are any TB multimorbidities mentioned in any of the following documents? Clinical guidelines Is Not Selected

And 7- In your country, are any TB multimorbidities mentioned in any of the following documents? Coordination management tools Is Not Selected

And 7- In your country, are any TB multimorbidities mentioned in any of the following documents? Other (please specify): Is Not Selected

Please answer the following questions **considering the TB service/ clinic you are based in.** We want to know how TB Multimorbidity is considered in TB services/ clinics ***in practice***.

Q16

9- Do you work in a TB clinic?

- ◯ Yes, I do work in a TB clinic
- ◯ No, I do not work in a TB clinic

Q19

10- Regarding screening, diagnosis and surveillance...

For each of the questions below, please specify the conditions considered in your TB service.

	None	Don't know / Unsure	HIV	Diabetes Mellitus	Hepatitis C virus	Hepatitis B virus	Depression	Anxiety
What conditions can the TB service **diagnose**? (has the **facilities and expertise**)	☐	☐	☐	☐	☐	☐	☐	☐
What conditions does the TB service **screen** for?	☐	☐	☐	☐	☐	☐	☐	☐
For what conditions does the TB service **routinely collect data**?	☐	☐	☐	☐	☐	☐	☐	☐
For what conditions does the TB service **report to the TB programme**?	☐	☐	☐	☐	☐	☐	☐	☐
For what conditions does the TB service **report to other disease control programme**?	☐	☐	☐	☐	☐	☐	☐	☐

Q20

11- Regarding treatment...

For each of the questions below, please specify the conditions considered in your TB service.

	None	Don't know / Unsure	HIV	Diabetes Mellitus	Hepatitis C virus	Hepatitis B virus	Depression	Anxiety
For what conditions is the TB service **able to start care**?	☐	☐	☐	☐	☐	☐	☐	☐
For what conditions does the TB service **offer maintenance care**?	☐	☐	☐	☐	☐	☐	☐	☐
For what conditions does the TB service **refer patients to a specialised service**?	☐	☐	☐	☐	☐	☐	☐	☐
For what conditions does the TB service **liaise with other services**?	☐	☐	☐	☐	☐	☐	☐	☐
For what conditions does the TB service **provide medication**?	☐	☐	☐	☐	☐	☐	☐	☐
For what conditions do TB service providers **receive training**?	☐	☐	☐	☐	☐	☐	☐	☐
What conditions are covered in **supervision, monitoring and evaluation**?	☐	☐	☐	☐	☐	☐	☐	☐

Q21

Display this question

If 11- Regarding treatment... For each of the questions below, please specify the conditions conside... - For what conditions does the TB service provide medication? Is Greater Than 0

And 11- Regarding treatment... For each of the questions below, please specify the conditions conside... None - For what conditions does the TB service provide medication? Is Not Selected

And 11- Regarding treatment... For each of the questions below, please specify the conditions conside... Don't know / Unsure - For what conditions does the TB service provide medication? Is Not Selected

11b- You mentioned that the TB service provides medication for some conditions. Is this medication provided for free?

	HIV	Diabetes Mellitus	Hepatitis C virus	Hepatitis B virus	Depression	Anxiety	Tobacco use	Alcoho use
Free	○	○	○	○	○	○	○	○
Not free	○	○	○	○	○	○	○	○

Q22

12- Please feel free to add any additional details...

Q23

Page 4 of 5

Import from library

Add new question

Add Block

Block 4

Q24

In the following questions we want to know more about your experience working in a TB service / clinic.

Q25 *

13- In your setting, and in your experience, what are the *three most common* conditions you find in people with TB?

- ☐ HIV
- ☐ Diabetes Mellitus
- ☐ Hepatitis C virus
- ☐ Hepatitis B virus
- ☐ Depression
- ☐ Anxiety
- ☐ Tobacco use
- ☐ Alcohol use
- ☐ Chronic renal failure
- ☐ * Other (1): ____
- ☐ * Other (2): ____
- ☐ * Other (3): ____

Q26 *

14- In your setting, and in your experience, what are the *three most concerning* conditions you find in people with TB?

- ☐ HIV
- ☐ Diabetes Mellitus
- ☐ Hepatitis C virus
- ☐ Hepatitis B virus
- ☐ Depression
- ☐ Anxiety
- ☐ Tobacco use
- ☐ Alcohol use
- ☐ Chronic renal failure
- ☐ * Other (1): ____
- ☐ * Other (2): ____
- ☐ * Other (3): ____

Q27

Display this question

If 9- Do you work in a TB clinic? Yes, I do work in a TB clinic Is Selected

15- For each of the conditions below, select the areas (diagnosis and/or treatment) you feel capable to perform.
Feel free to add any other conditions that you consider relevant in the context of TB multimorbidity.

	Diagnose	Treat
HIV	☐	☐
Diabetes Mellitus	☐	☐
Hepatitis C virus	☐	☐
Hepatitis B virus	☐	☐
Depression	☐	☐
Anxiety	☐	☐
Tobacco use	☐	☐
Alcohol use	☐	☐
Chronic renal failure	☐	☐
* Other (1): ______	☐	☐
* Other (2): ______	☐	☐
* Other (3): ______	☐	☐

Q28

Page 5 of 5

Import from library   Add new question

Add Block

End of Survey

Thank you for completing this survey. Your responses have now been submitted.

If you want us to send you a brief report with the results once we have them, please contact us at alexander.jarde@york.ac.uk.

Please visit the TB Multimorbidity Network's website to learn more about our group and our research.

We want to reach as many people of interest as possible. If you know someone else who might meet our inclusion criteria, please forward them the email invitation that you received or just send them the following link to

this survey: https://york.qualtrics.com/jfe/form/SV_24DRqlt0JMET5ki?SRC=EoS&Q_Language=EN

Thank you again for your collaboration.
