# Supplementary material for: Addressing TB multimorbidity in policy and practice: An exploratory survey of TB providers in 27 high-TB burden countries
Source: PLOS Glob Public Health. 2022 Dec 7;2(12):e0001205. doi: 10.1371/journal.pgph.0001205 (PMC10022227; doi:10.1371/journal.pgph.0001205)

# Tuberculosis Multimorbidity Survey

## Respondent’s characteristics

In the first section we collected data about the respondent: where they worked, their role in the health care system, etc.

*What country do you work in?*

We included responses from 27 countries, with four countries (Brazil [116], India [77], the Philippines [62], and China [39]) covering two thirds of all responses (66%). We had 20 or more responses from Brazil, India, Philippines, China, Nigeria, and Uganda, and only one response from the Central African Republic, Indonesia, Liberia, Namibia, and the Russian Federation (Figure A).

Figure A: Number of responses by country

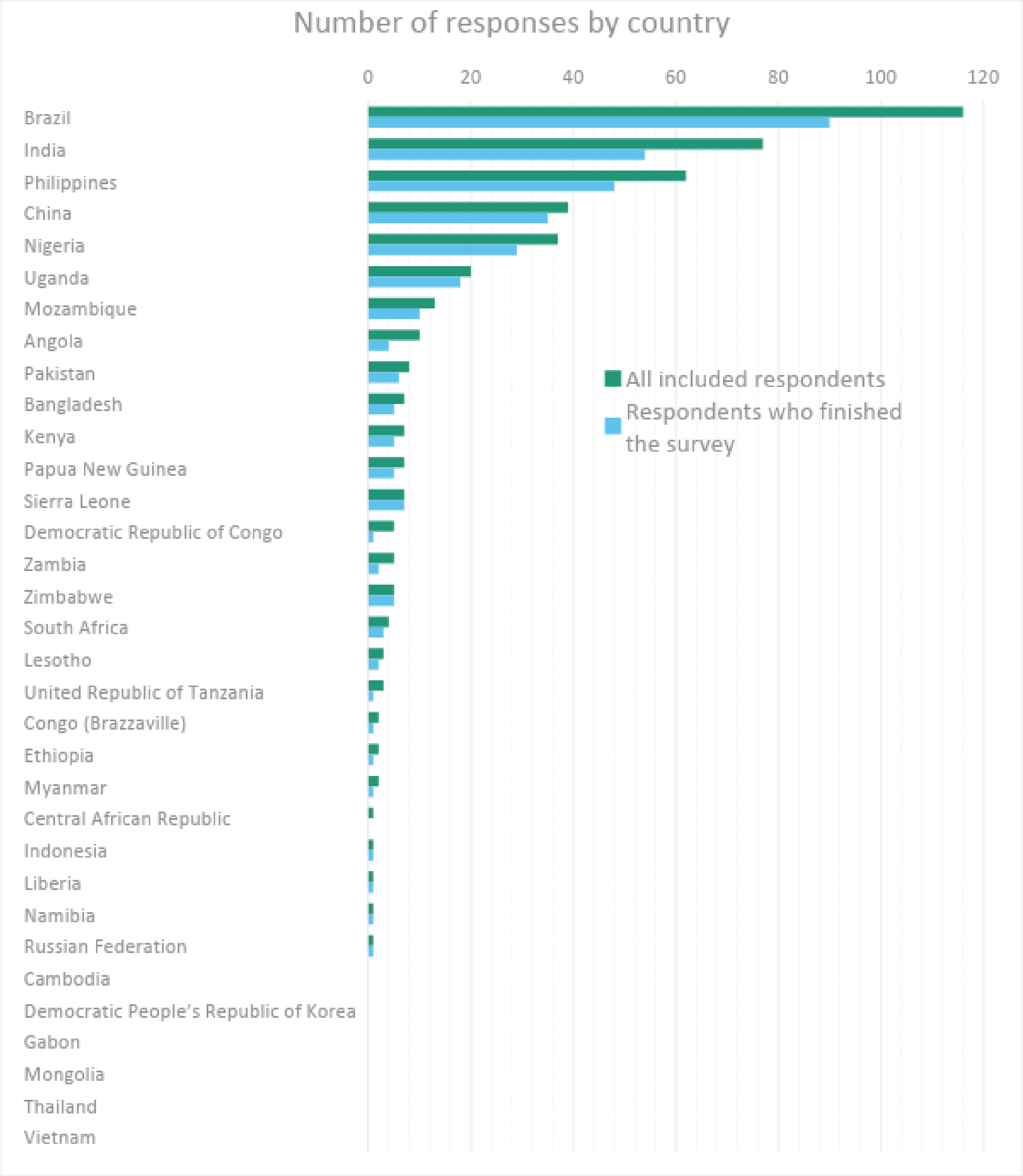


*What statement best describes your position?*

About one third of respondents (35%) described themselves as 'TB programme manager/ supervisor/ coordinator' (*TBP manager*), and a similar number (36%) described themselves as working with people with TB in primary, secondary, or tertiary health care. Half of the remaining third of responses were from 'UNION/ WHO/ other NGO's consultant/ advocate/ advisor' (*Consultant*, 14%) and 15% of respondents described their position as 'other' (Figure B).

Figure B: What statement best describes your position?

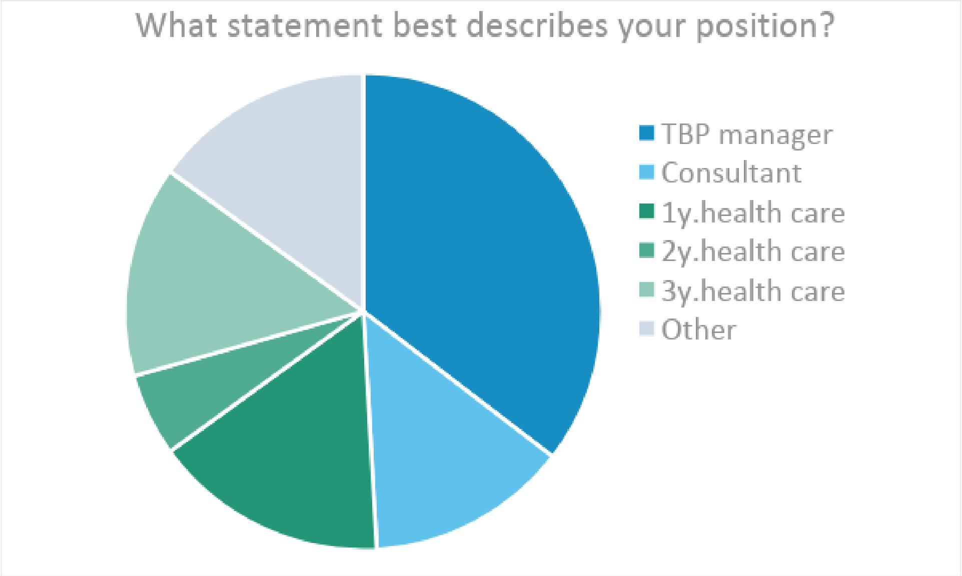


*3b- Do you work at a DOTS clinic?*

Those respondents who answered that they worked in health care were asked if they worked in a DOTS clinic. About half of them (52%) answered 'Yes' (Table B).

Table B: Do you work at a DOTS clinic? (only asked to health care workers)

| | Yes | No | NR | *Total* |
|---|---|---|---|---|
| **Primary health care** | 39 | 30 | 2 | *71* |
| **Secondary health care** | 10 | 14 | 1 | *25* |
| **Tertiary health care** | 34 | 27 | 3 | *64* |
| **Total** | 83 | 71 | 6 | *160* |

NR: No response

*5- What is your main role in the organisation / TB service?*

When asked about their role in the organisation / TB service, 162 (36%) described their role as 'Clinician/ TB healthcare professional', 105 (24%) described their role as 'Service manager' and 78 (17%) as 'Advocacy or advisory'. Interestingly, the overlap between position and role indicates that not all health care workers have a clinical role, nor all TBP managers describe their role as a service manager (Table C).

Table C: What is your main role in the organisation / TB service?

| | TBP manager | Consultant | Primary health care | Secondary health care | Tertiary health care | Other | Total |
|---|---|---|---|---|---|---|---|
| **Service manager** | 81 | 9 | 2 | 1 | 4 | 8 | **105 (24%)** |

| | | | | | | | |
|---|---|---|---|---|---|---|---|
| **Clinician/ TB healthcare professional** | 28 | 8 | 47 | 20 | 53 | 6 | **162 (36%)** |
| **Advocacy / advisory** | 19 | 33 | 9 | 1 | 4 | 12 | **78 (17%)** |
| **Other** | 29 | 8 | 12 | 3 | 2 | 39 | **93 (21%)** |
| **NR** | 1 | 3 | 1 | 0 | 1 | 2 | **8 (2%)** |

NR: No response; TBP: TB programme

### *6a- What type of TB service provider do you work at?*

Those respondents who answered that they worked in health care (primary, secondary or tertiary) were asked about the type of service provider. Half of them worked in the public sector and most of those working in the private sector did so in a not-for-profit organisation, mostly in primary health care (Table D).

Table D: What type of TB service provider do you work at? (only asked to health care workers)

| Type of service provider | Primary health care | Secondary health care | Tertiary health care | Total |
|---|---|---|---|---|
| **Public** | 45 | 18 | 54 | **117 (52%)** |
| **Private (not-for-profit)** | 18 | 4 | 6 | **28 (12%)** |
| **Private (for-profit)** | 3 | 1 | 3 | **7 (3%)** |
| **Other** | 4 | 1 | 0 | **5 (2%)** |
| **NR** | 1 | 1 | 1 | **70 (31%)** |

NR: No response

### *6b- At what level are you working?*

Those respondents who answered that they worked as TB programme manager/ supervisor/ coordinator, or who said they were a UNION/ WHO/ other NGO's consultant/ advocate/ advisor were asked at what level they were working. Around one third of these respondents answered that they worked at the national and/or at the district level (multiple answers were allowed), with very few working at an international level (Figure C).

Figure C: At what level are you working (only asked to TBP managers, consultants, etc.)

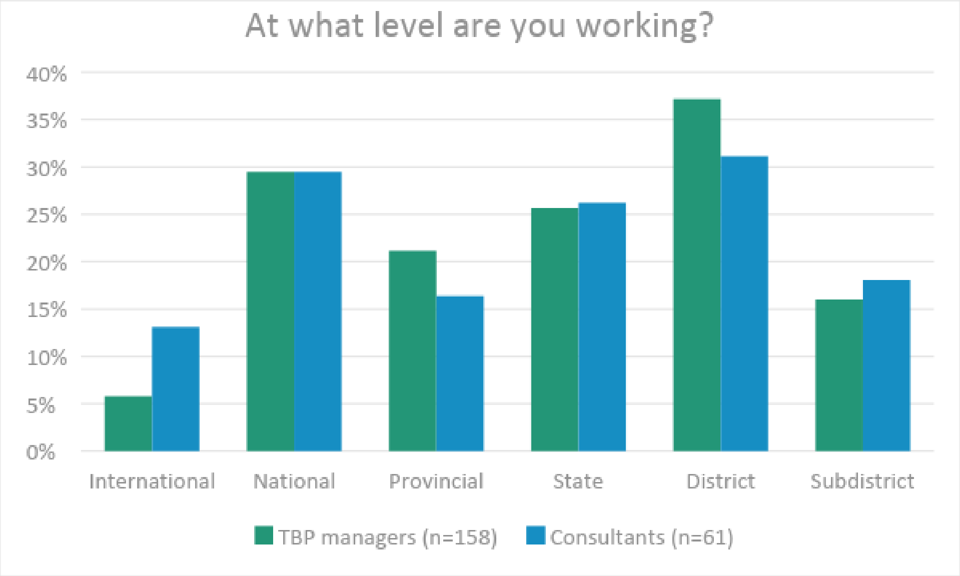


## Non-respondent's characteristics

While it is not possible to know the characteristics of those who did not respond because of the anonymity of those who did, we can look at the characteristics of those who started the survey but did not answer any of the questions beyond the participant's characteristics ones.

### *What country do you work in?*

While the countries with most respondents were also among those with most people who only answered to the initial questions, the number of such unfinished surveys is surprisingly low for India, and unexpectedly high for the Philippines.

### Figure D: Number of people who only answered to initial questions, by country

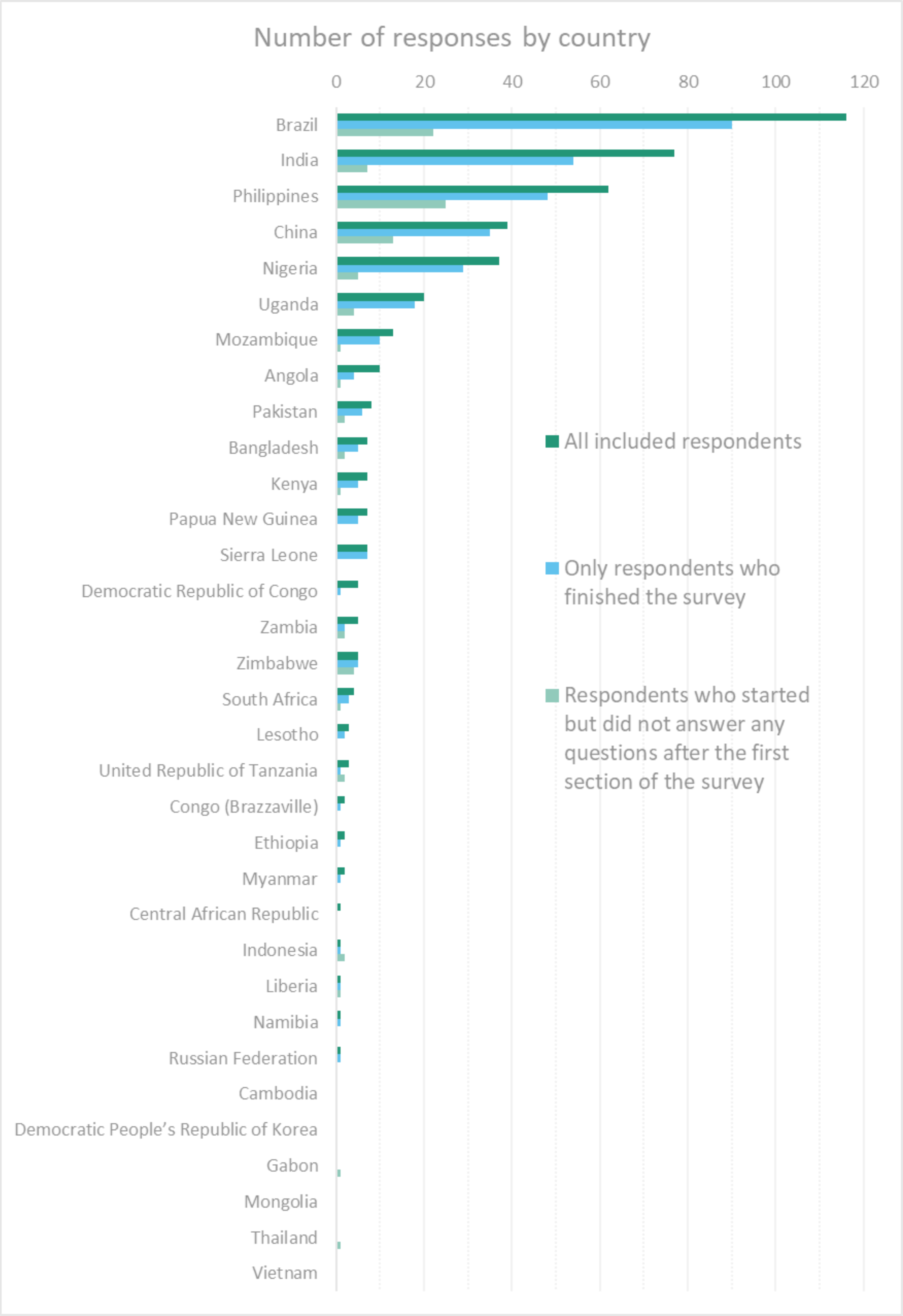


#### *What statement best describes your position?*

The proportion of respondents describing themselves as 'TB programme manager/supervisor/coordinator' (*TBP manager*, 37.1%) and as working with people with TB

in primary, secondary, or tertiary health care (42.3%), or as ‘Other’ (14.4%) are not very different from the responses given by people who answered a larger part of the survey. Only the proportion of *Consultant*s is a bit lower.

Figure E: What statement best describes your position?

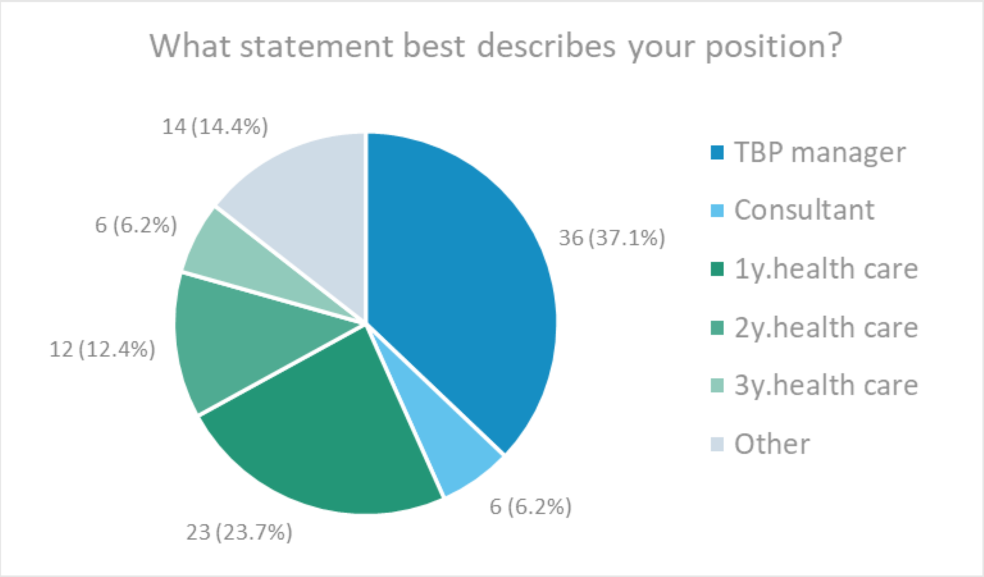


*3b- Do you work at a DOTS clinic?*

Those respondents who answered that they worked in health care were asked if they worked in a DOTS clinic. Close to half of those who did not continued with the survey (44%) answered ‘Yes’ (Table E).

Table E: Do you work at a DOTS clinic? (only asked to health care workers)

| | Yes | No | NR | *Total* |
|---|---|---|---|---|
| **Primary health care** | 14 | 8 | 1 | *23* |
| **Secondary health care** | 2 | 10 | 0 | *12* |
| **Tertiary health care** | 2 | 4 | 0 | *6* |
| **Total** | 18 | 22 | 1 | *41* |

NR: No response

*5- What is your main role in the organisation / TB service?*

Close to half (46%) of those who started the survey but did not answer any questions beyond respondent’s characteristics did not answer this question, which might reflect their lack of engagement.

| | TBP manager | Consultant | Primary health care | Secondary health care | Tertiary health care | Other | Total |
|---|---|---|---|---|---|---|---|
| **Service manager** | 8 | 2 | 2 | 0 | 0 | 2 | **14 (14%)** |

| | | | | | | | |
|---|---|---|---|---|---|---|---|
| **Clinician/ TB healthcare professional** | 7 | 0 | 4 | 3 | 4 | 2 | **20 (21%)** |
| **Advocacy / advisory** | 1 | 0 | 2 | 1 | 0 | 1 | **5 (5%)** |
| **Other** | 2 | 2 | 2 | 1 | 0 | 6 | **13 (13%)** |
| **NR** | 18 | 2 | 13 | 7 | 2 | 3 | **45 (46%)** |

NR: No response; TBP: TB programme

*6a- What type of TB service provider do you work at?*

The majority (65%) of those who started the survey but did not answer any questions beyond respondent's characteristics did not answer this question, which might reflect their lack of engagement or that they had left the survey already.

Table F: What type of TB service provider do you work at? (only asked to health care workers)

| Type of service provider | Primary health care | Secondary health care | Tertiary health care | Total |
|---|---|---|---|---|
| **Public** | 6 | 2 | 4 | **0 (22%)** |
| **Private (not-for-profit)** | 3 | 2 | 0 | **0 (9%)** |
| **Private (for-profit)** | | | | **(0%)** |
| **Other** | 1 | 1 | 0 | **0 (4%)** |
| **NR** | 13 | 7 | 2 | **14 (65%)** |

NR: No response

*6b- At what level are you working?*

Only 21 TBP managers and 4 Consultants reached this question but who did not answer any other question.

Figure F: At what level are you working (only asked to TBP managers, consultants, etc.)

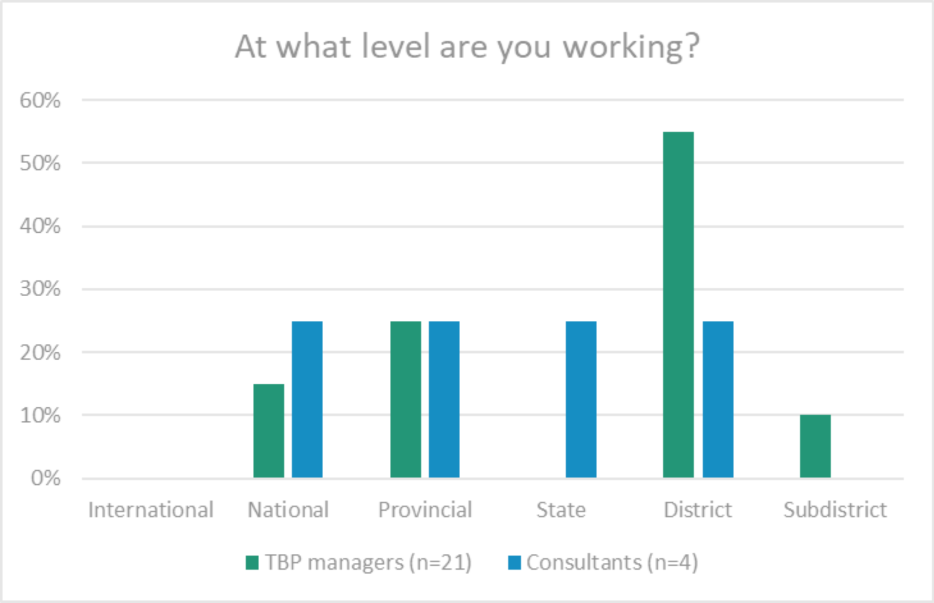

Supplement: S3 Appendix — (PDF) [file pgph.0001205.s003.pdf]
