# Supplementary material for: Addressing TB multimorbidity in policy and practice: An exploratory survey of TB providers in 27 high-TB burden countries
Source: PLOS Glob Public Health. 2022 Dec 7;2(12):e0001205. doi: 10.1371/journal.pgph.0001205 (PMC10022227; doi:10.1371/journal.pgph.0001205)
Supplement: S1 File — (PDF) [file pgph.0001205.s004.pdf]

Tuberculosis Multimorbidity Survey

Report of descriptive results

Tuberculosis Multimorbidity Survey

Introduction *3*

Methods *3*

Sampling strategy 3

Inclusion and exclusion criteria 4

Data collection 4

Data management and analysis 5

Results *5*

Respondent's characteristics 5

What country do you work in? 5

What statement best describes your position? 6

3b- Do you work at a DOTS clinic? 7

5- What is your main role in the organisation / TB service? 7

6a- What type of TB service provider do you work at? 8

6b- At what level are you working? 8

Non-respondent's characteristics 9

What country do you work in? 9

What statement best describes your position? 10

3b- Do you work at a DOTS clinic? 11

5- What is your main role in the organisation / TB service? 11

6a- What type of TB service provider do you work at? 12

6b- At what level are you working? 12

TB Multimorbidity in theory - Policies, strategic plans, and clinical guidelines. 12

7- In your country, are any TB multimorbidities mentioned in any of the following documents? 13

8- What conditions are mentioned, and what are clinicians asked to do? 15

8b- What conditions are mentioned, and what are clinicians asked to do? - Overview 18

TB Multimorbidity in practice – Screening, diagnosis, surveillance and treatment... 20

9- Do you work in a TB clinic? 20

10- Regarding screening, diagnosis and surveillance... 20

11- Regarding treatment... 22

10+11 Overview of screening and treatment 24

11b- Is medication provided for free? 25

Respondents' experience working in a TB service / clinic 26

13- In your setting, and in your experience, what are the three most common conditions you find in people with TB? 27

14- In your setting, and in your experience, what are the three most concerning conditions you find in people with TB? 27

15- For each of the conditions below, select the areas (diagnosis and/or treatment) you feel capable to perform 28

Overview: TB Multimorbidity in theory, in practice, and perceived capability... 29

Discussion *30*

Strengths and limitations 31

Conclusion 31

References *32*

Introduction

In 2018, tuberculosis (TB) was the leading cause of mortality (1.4 million deaths) from a single infectious disease globally (1). Multimorbidity, defined as the co-occurrence of two or more chronic conditions in a single individual at one point in time, is also a growing global concern (2). Among people with TB, the prevalence of various comorbidities has been estimated in different meta-analyses, which report about half of TB patients in LMIC have depression (3), about one third in Sub-Saharan Africa have HIV (4) and around 15% have diabetes (5). Furthermore, people with TB and at least one comorbidity have been found to have significantly worse treatment outcomes than people with TB only (6–10). While there is growing support to manage TB multimorbidity with an integrated patient-centred approach (e.g. (11)), it is unclear to what extent these other mental and physical health comorbidities are managed by TB services. Our aim is to better understand if and how TB multimorbidity is currently considered and addressed in high-TB burden countries (12).

Our key objectives were, overall and for each country, to:

1) understand the extent to which TB multimorbidity is recognised and addressed in policy, management and services within TB healthcare.
2) understand which specific comorbidities are being considered in TB healthcare.
3) to identify potential gaps in service provision for TB multimorbidity.

Our research questions were:

1. At the management level, what proportion of TB policies and strategic plans, management tools (checklists, guides, etc.) and clinical guidelines take TB multimorbidity into account in TB high burden countries?
2. At the level of TB service provision, what proportion of TB service programs take TBMM into account in terms of screening, diagnosis, surveillance, treatment, and management in TB high burden countries?
3. What TB comorbidities are being considered more often in TB programmes and services in each individual country?
4. What comorbidities are considered by TB service providers to be the most relevant to address?
5. Are there any discrepancies between policies, practice and perceived relevance?

Methods

In this cross-sectional study we conducted a multinational online survey to find out the extent to which TB services consider TB multimorbidity in their policies, management, guidelines and services. This study was approved by the Research Governance Committee of the University of York.

Sampling strategy

We used a purposive sampling technique to try to identify a variety of key respondents in management, advisory, and clinical positions at different levels of the public healthcare system and the private sector in each country.

We used a variety of methods to try to directly or indirectly reach and engage the widest range of potential respondents. On one hand, we searched for contact details of potential respondents:

a) For each country, we identified organizations from the Stop TB Partnership Partner's Directory (http://www.stoptb.org/partners/) and extracted their contact details.

b) For each country, we did online searches (using the Google search engine) with the terms 'TB programme' and the country name to try to identify contact details of TB programme managers and NGO's.

c) We asked members of the Tuberculosis Multimorbidity Network (https://www.impactsouthasia.com/tbmm/) and other colleagues involved in TB programmes about contact details of potential respondents.

On the other hand, we searched for contact details of potential *sources* of contacts. That is: people who we thought could provide us with potential respondent's contact details or who we believed had a network of contacts to whom they could forward our invitation email to. These included:

d) Representatives of The Union and the World Health Organization

e) For each country, we searched for research articles using the keywords 'tuberculosis' and the country name and identified researchers who had published articles on the topic, as we assumed that they would have a network of contacts in the country.

Finally, we also promoted the TBMM Survey on twitter, inviting potential respondents to participate, and encouraged survey participants to forward our invitation to take the survey to other potential participants (snowball sampling).

The email invite contained a brief description of the study and a link to the online survey, with a reminder email sent halfway through the data collection period. The first page of the survey also included a link to the participant information sheet detailing the goals of the study, the confidentiality of the data collected, and their right to leave the study at any point in time. Continuing with the survey was taken as consent for participation.

We had planned to include in the survey all 30 countries (mostly in Africa and Asia) classified by the WHO as a high-burden TB country (12). However, in June 2021, the WHO updated the list of high burden countries for Tuberculosis, adding Gabon, Mongolia and Uganda to the list, and removing Cambodia, the Russian Federation and Zimbabwe (13). Given that this change happened only weeks before we launched our survey, we decided to add the new countries, but without excluding Cambodia, Russia and Zimbabwe.

Inclusion and exclusion criteria

We included any TB service providers in the primary, secondary and tertiary health care, in either the public or the private (for profit or not for profit) sector. Respondents providing information on these services could have a management, clinician or advisory/advocacy role. In addition, TB program managers, supervisors and coordinators at the national, provincial, regional and district level were also included.

Data collection

We prepared an online survey (using Qualtrix) which covered the following topics: 1) Questions about the respondent's characteristics (country, position, etc.); 2) questions related to policies, strategic plans, and clinical guidelines; 3) questions related to services and practice; and 4) questions regarding the respondent's experience and concerns. All the questions, including skipping rules for individual items, can be seen in the supplementary pdf file.

French and Portuguese versions of the survey were also prepared and linked to in invitations sent to potential respondents in French- and Portuguese-speaking countries, respectively.

Data management and analysis

We summarised quantitative and qualitative responses using descriptive analyses and identifying themes, respectively. These were planned to be presented by country, by type of service provider (based on their stated position), and overall. However, in light of the heterogeneity in the respondents for each country we decided to not pool all respondents together (for overall)

We had limited short text responses for some of the questions, which we planned to summarise and collate by themes.

Results

The first email invitations were sent out on the 8th of August 2021, with a follow-up reminder sent two weeks later. Responses were collected until 18th of October 2021, at which point we stopped the survey and downloaded the response data to be analysed. In this period, 543 people started the survey, although 97 (17.86%) did not make it past the respondent characteristics questions and their data was excluded, leaving 446 included responses, 76% of which (337) were complete (Table 1).

Table 1: Number of incomplete and complete surveys

Response progress	Number of responses	% of Total	% of included
**Incomplete** (not past respondent characteristics)	97	17.86%	(excluded)
**Incomplete** (more than respondent characteristics)	109	20.08%	24.44%
**Complete**	337	62.06%	75.56%
**Total included**	446	82.14%	100%

Respondent's characteristics

In the first section we collected data about the respondent: where they worked, their role in the health care system, etc.

What country do you work in?

We included responses from 27 countries, with four countries (Brazil [116], India [77], the Philippines [62], and China [39]) covering two thirds of all responses (66%). We had 20 or more responses from Brazil, India, Philippines, China, Nigeria, and Uganda, and only one response from the Central African Republic, Indonesia, Liberia, Namibia, and the Russian Federation (Figure 1).

Figure 1: Number of responses by country

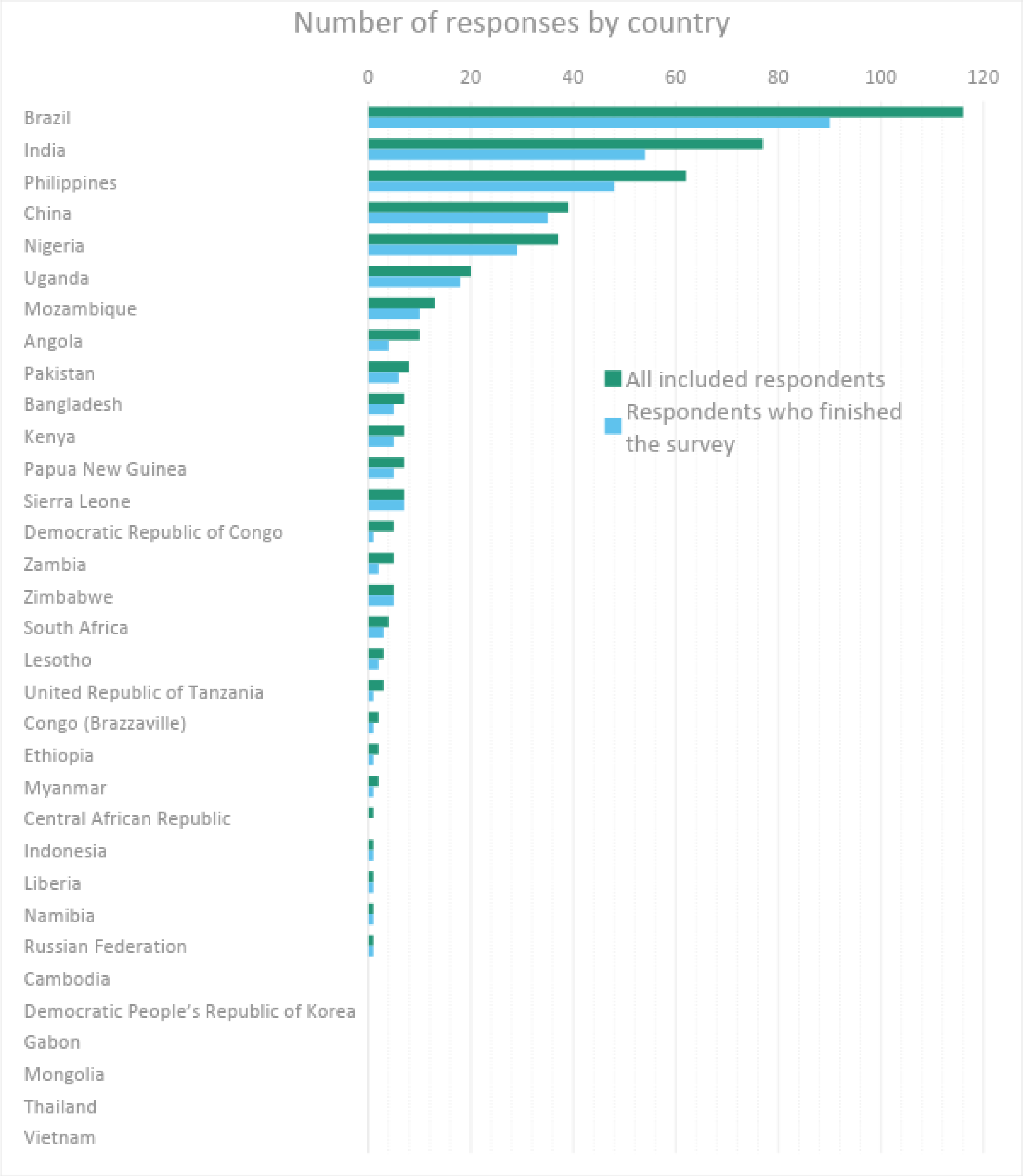

What statement best describes your position?

About one third of respondents (35%) described themselves as 'TB programme manager/ supervisor/ coordinator' (*TBP manager*), and a similar number (36%) described themselves as working with people with TB in primary, secondary, or tertiary health care. Half of the remaining third of responses were from 'UNION/ WHO/ other NGO's consultant/ advocate/ advisor' (*Consultant*, 14%) and 15% of respondents described their position as 'other' (Figure 2).

Figure 2: What statement best describes your position?

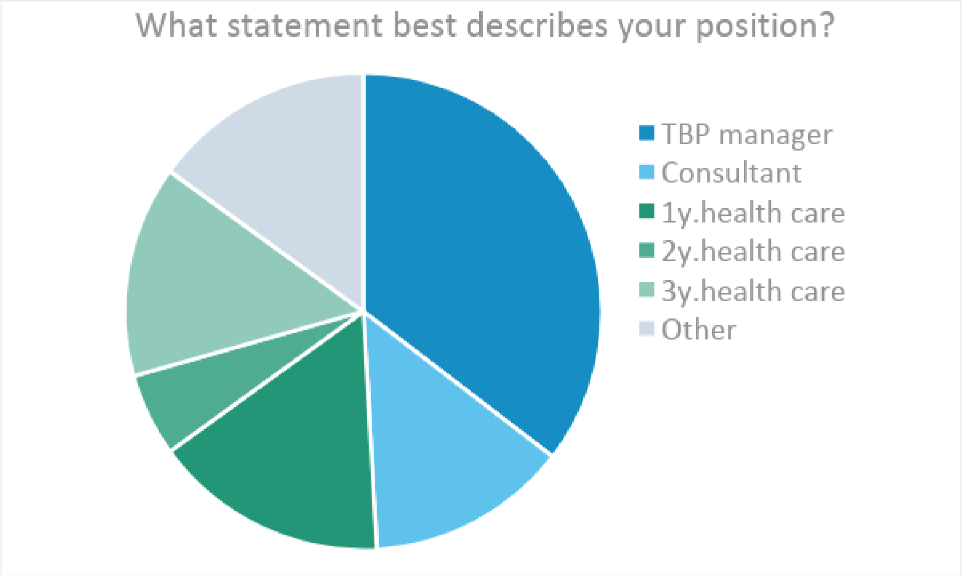

3b- Do you work at a DOTS clinic?

Those respondents who answered that they worked in health care were asked if they worked in a DOTS clinic. About half of them (52%) answered 'Yes' (Table 2).

Table 2: Do you work at a DOTS clinic? (only asked to health care workers)

	Yes	No	NR	*Total*
Primary health care	39	30	2	*71*
Secondary health care	10	14	1	*25*
Tertiary health care	34	27	3	*64*
Total	83	71	6	*160*

NR: No response

5- What is your main role in the organisation / TB service?

When asked about their role in the organisation / TB service, 162 (36%) described their role as 'Clinician/ TB healthcare professional', 105 (24%) described their role as 'Service manager' and 78 (17%) as 'Advocacy or advisory'. Interestingly, the overlap between position and role indicates that not all health care workers have a clinical role, nor all TBP managers describe their role as a service manager (Table 3).

Table 3: What is your main role in the organisation / TB service?

	TBP manager	Consultant	Primary health care	Secondary health care	Tertiary health care	Other	Total
Service manager	81	9	2	1	4	8	105 (24%)

**Clinician/ TB healthcare professional**	28	8	47	20	53	6	**162 (36%)**
**Advocacy / advisory**	19	33	9	1	4	12	**78 (17%)**
**Other**	29	8	12	3	2	39	**93 (21%)**
**NR**	1	3	1	0	1	2	**8 (2%)**

NR: No response; TBP: TB programme

6a- What type of TB service provider do you work at?

Those respondents who answered that they worked in health care (primary, secondary or tertiary) were asked about the type of service provider. Half of them worked in the public sector and most of those working in the private sector did so in a not-for-profit organisation, mostly in primary health care (Table 4).

Table 4: What type of TB service provider do you work at? (only asked to health care workers)

Type of service provider	Primary health care	Secondary health care	Tertiary health care	Total
**Public**	45	18	54	**117 (52%)**
**Private (not-for-profit)**	18	4	6	**28 (12%)**
**Private (for-profit)**	3	1	3	**7 (3%)**
**Other**	4	1	0	**5 (2%)**
**NR**	1	1	1	**70 (31%)**

NR: No response

6b- At what level are you working?

Those respondents who answered that they worked as TB programme manager/ supervisor/ coordinator, or who said they were a UNION/ WHO/ other NGO's consultant/ advocate/ advisor were asked at what level they were working. Around one third of these respondents answered that they worked at the national and/or at the district level (multiple answers were allowed), with very few working at an international level (Figure 3).

Figure 3: At what level are you working (only asked to TBP managers, consultants, etc.)

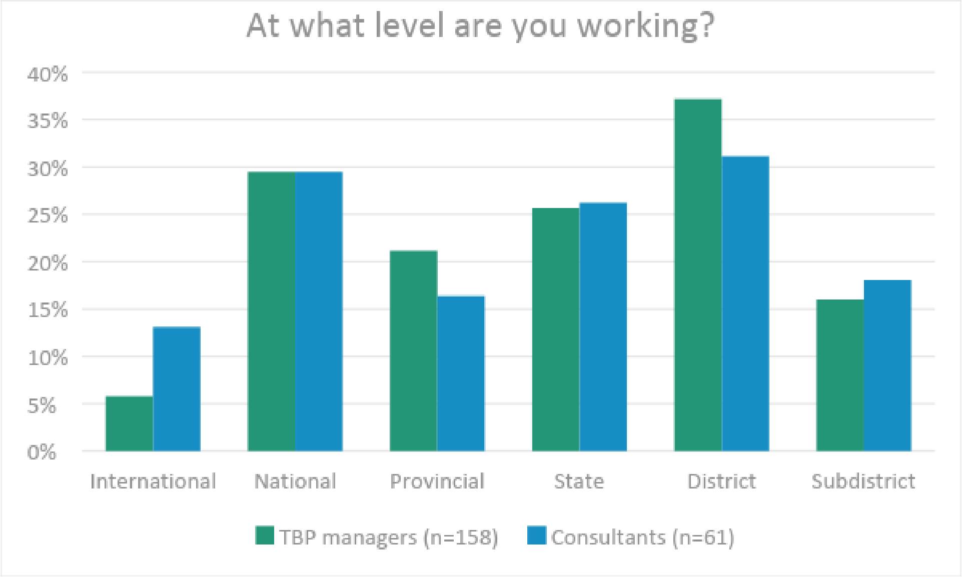

Non-respondent's characteristics

While it is not possible to know the characteristics of those who did not respond because of the anonymity of those who did, we can look at the characteristics of those who started the survey but did not answer any of the questions beyond the participant's characteristics ones.

What country do you work in?

While the countries with most respondents were also among those with most people who only answered to the initial questions, the number of such unfinished surveys is surprisingly low for India, and unexpectedly high for the Philippines.

Figure 4: Number of people who only answered to initial questions, by country

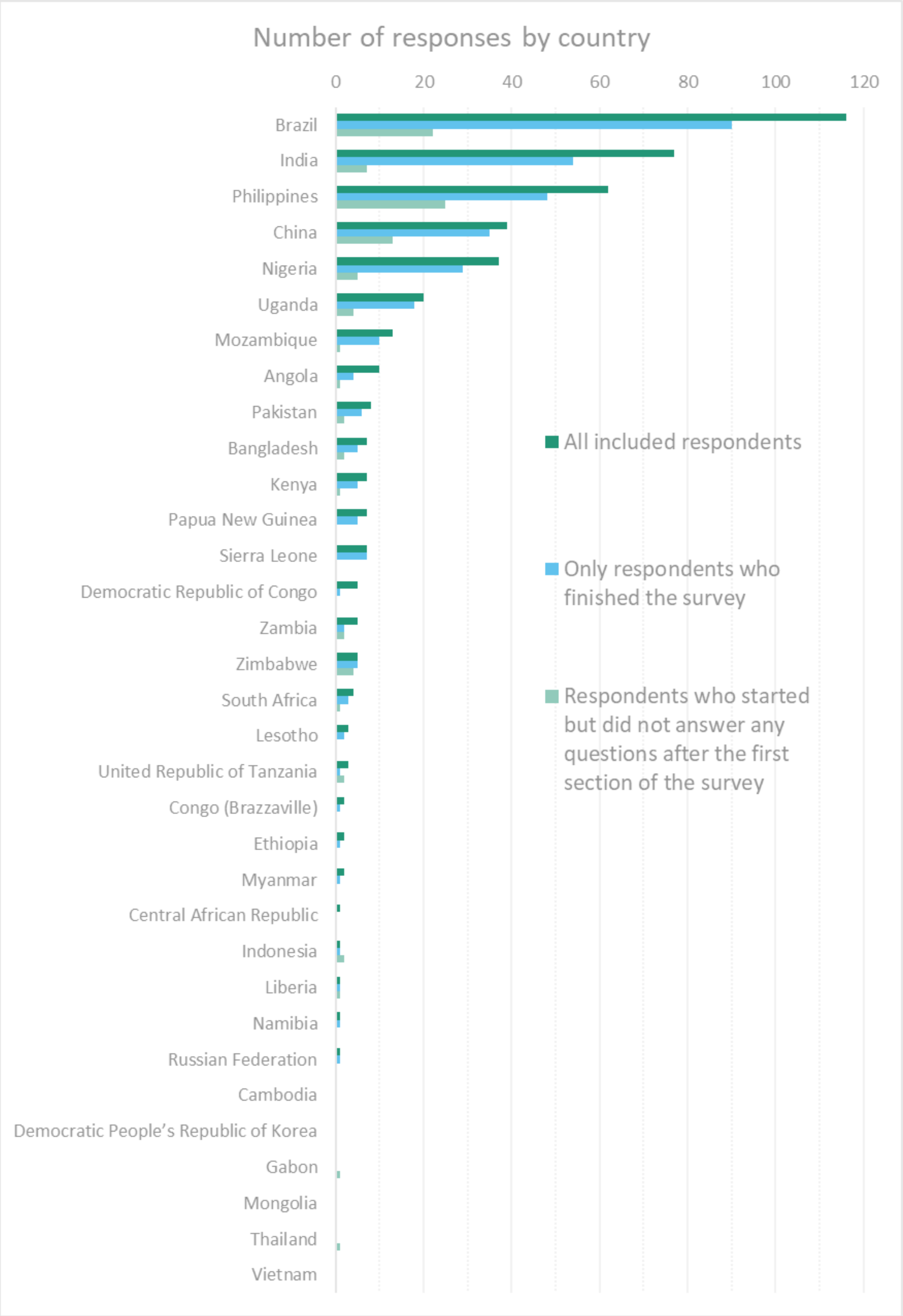

What statement best describes your position?

The proportion of respondents describing themselves as 'TB programme manager/supervisor/coordinator' (*TBP manager*, 37.1%) and as working with people with TB

in primary, secondary, or tertiary health care (42.3%), or as ‘Other’ (14.4%) are not very different from the responses given by people who answered a larger part of the survey. Only the proportion of *Consultant*s is a bit lower.

Figure 5: What statement best describes your position?

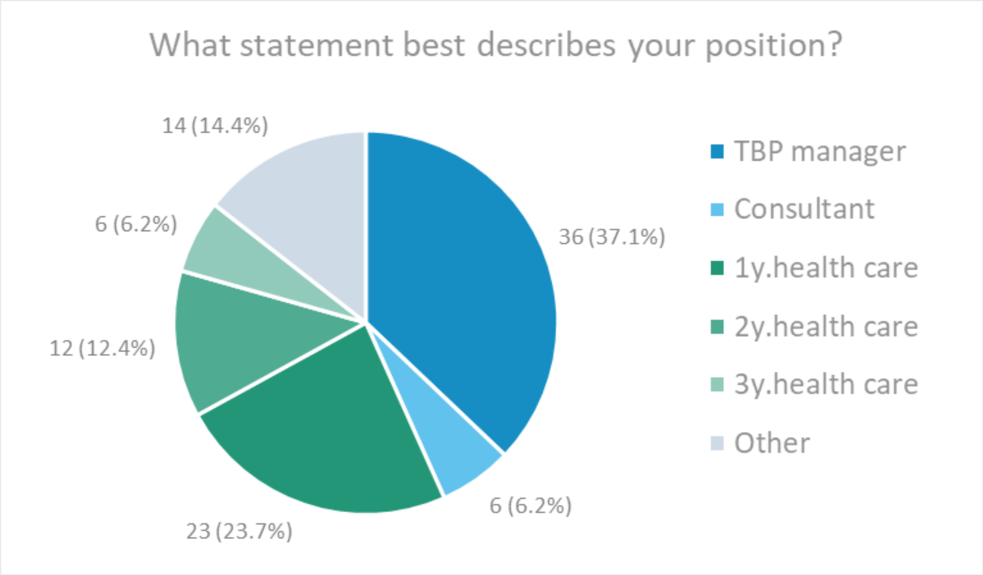

3b- Do you work at a DOTS clinic?
Those respondents who answered that they worked in health care were asked if they worked in a DOTS clinic. Close to half of those who did not continued with the survey (44%) answered ‘Yes’ (Table 5).

Table 5: Do you work at a DOTS clinic? (only asked to health care workers)

	Yes	No	NR	*Total*
Primary health care	14	8	1	*23*
Secondary health care	2	10	0	*12*
Tertiary health care	2	4	0	*6*
Total	18	22	1	*41*

NR: No response

5- What is your main role in the organisation / TB service?
Close to half (46%) of those who started the survey but did not answer any questions beyond respondent’s characteristics did not answer this question, which might reflect their lack of engagement.

	TBP manager	Consultant	Primary health care	Secondary health care	Tertiary health care	Other	Total
Service manager	8	2	2	0	0	2	14 (14%)

Clinician/ TB healthcare professional	7	0	4	3	4	2	20 (21%)
Advocacy / advisory	1	0	2	1	0	1	5 (5%)
Other	2	2	2	1	0	6	13 (13%)
NR	18	2	13	7	2	3	45 (46%)

NR: No response; TBP: TB programme

6a- What type of TB service provider do you work at?

The majority (65%) of those who started the survey but did not answer any questions beyond respondent's characteristics did not answer this question, which might reflect their lack of engagement or that they had left the survey already.

Table 6: What type of TB service provider do you work at? (only asked to health care workers)

Type of service provider	Primary health care	Secondary health care	Tertiary health care	Total
Public	6	2	4	0 (22%)
Private (not-for-profit)	3	2	0	0 (9%)
Private (for-profit)				(0%)
Other	1	1	0	0 (4%)
NR	13	7	2	14 (65%)

NR: No response

6b- At what level are you working?

Only 21 TBP managers and 4 Consultants reached this question but who did not answer any other question.

Figure 6: At what level are you working (only asked to TBP managers, consultants, etc.)

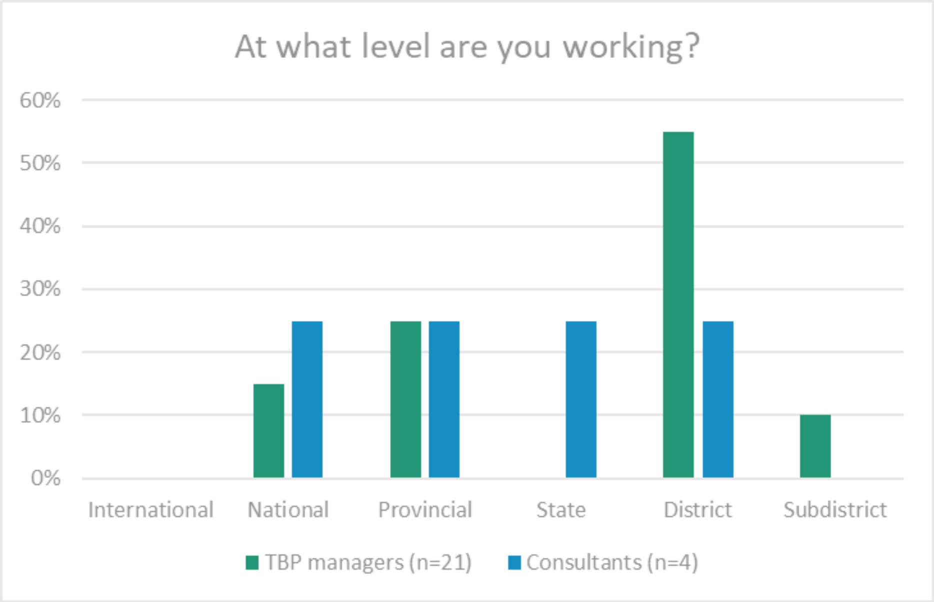

TB Multimorbidity in theory - Policies, strategic plans, and clinical guidelines.

In this section of the survey, we were interested in collecting data about how TB Multimorbidity was taken into account in official documents, such as policies, strategic plans, clinical guidelines, etc. We wanted to contrast the information collected in this section with what was actually happening in practice (next section).

For many of the questions in this and following sections we asked about a list of prespecified comorbidities, which were selected based on previous research and input from clinical experts. However, we also allowed respondents to add any conditions they thought were important. Based on these open-text fields, Covid-19, drug use, hypertension and malnutrition were also considered in the analyses.

With data from over a dozen conditions, this report will often focus on the 5 most relevant ones (based on which conditions the respondents considered most common and concerning): HIV, DM, tobacco use, alcohol use, and depression (see section on Respondents' experience working in a TB service / clinic below).

7- In your country, are any TB multimorbidities mentioned in any of the following documents?

In this first question we wanted to know if any TB multimorbidities were mentioned at all in any documents. As can be seen in Figure 4, 96% of respondents reported that TB multimorbidity was mentioned in at least one document. It is worth mentioning that in the countries where one or two respondents answered that none of the documents considered TB multimorbidity, other respondents from those same countries said the contrary.

Figure 7: In your country, are any TB multimorbidities mentioned in any of the following documents?

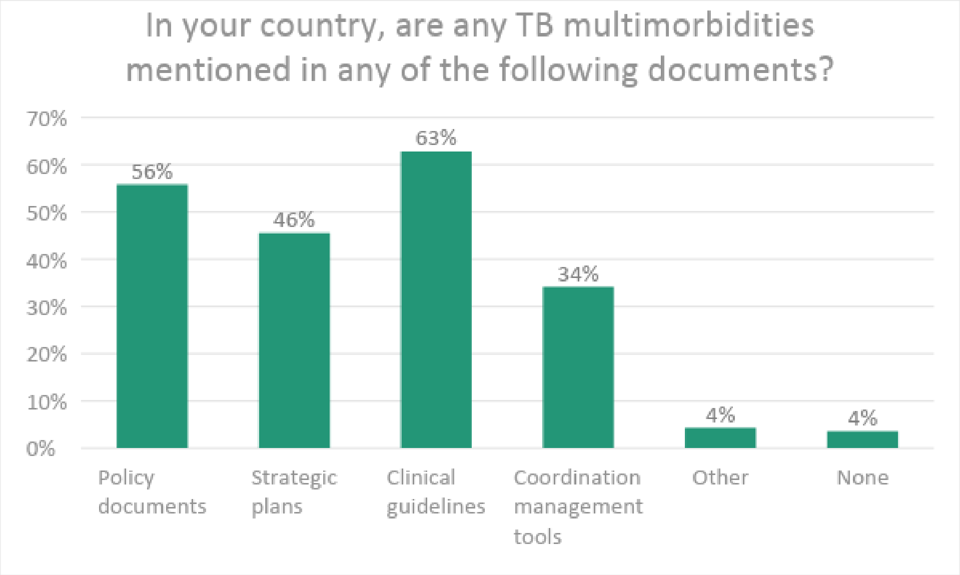

When we look at the responses by position, it is interesting to see that the % of positive responses is highest among TBP managers with regards to policy documents and strategic plans, while secondary and tertiary health care workers (but not primary health care workers) have a higher % of positive response regarding clinical guidelines. This could reflect how each group might be more familiar with documents that are more relevant in their position (Figure 5).

Considering responses by country, we can see that in all countries, the majority (if not all) of the respondents indicated that TB multimorbidity was considered in at least one of the mentioned documents (Figure 6).

Figure 8: In your country, are any TB multimorbidities mentioned in any of the following documents? (by position)

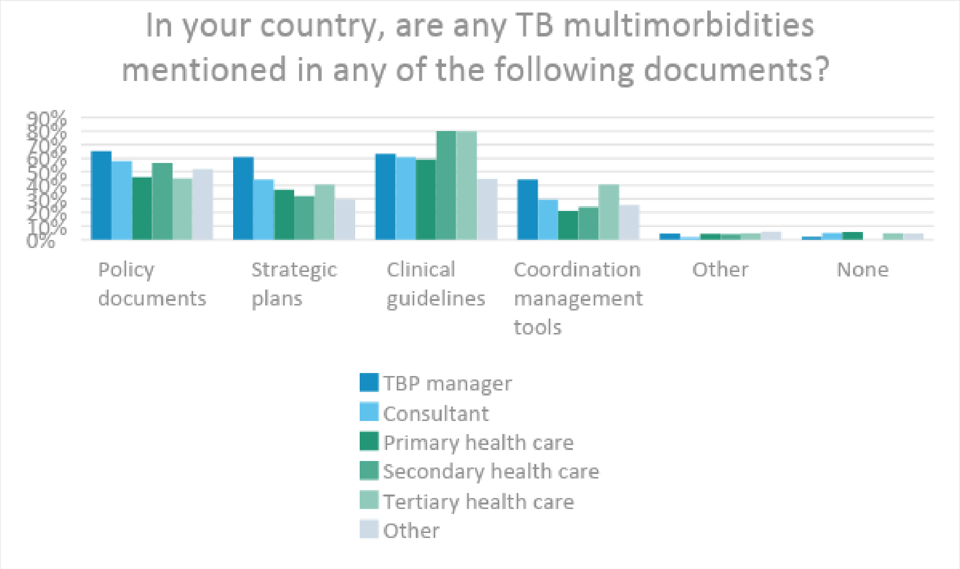

Figure 9: For each country (number of responses), the % of responses where TB multimorbidities are mentioned in at least one document

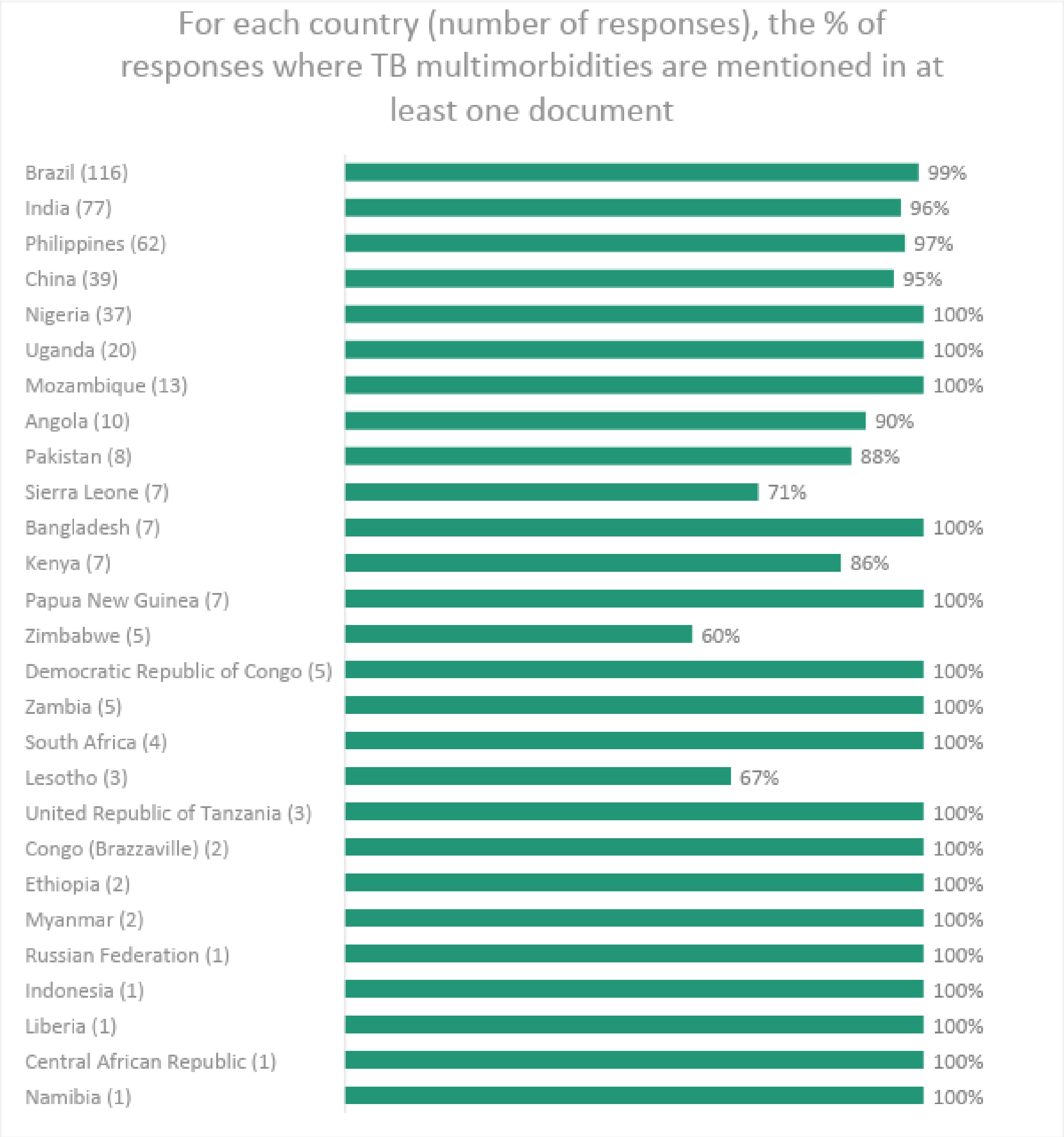

8- What conditions are mentioned, and what are clinicians asked to do?

In the following tables we describe the results for the most common and concerning conditions (according to the respondents: HIV, DM, tobacco use, alcohol use, and depression) for countries with 5 or more responses. The complete tables (with all countries and all conditions) can be found in the Supplement. We will consider that a condition is considered in policy documents and guidelines if 50% or more of the respondents say so.

HIV - HIV in people with TB is considered in policy documents and guidelines in all countries except Angola, where clinicians are not asked to neither prevent, screen or treat the condition. In Pakistan, clinicians are asked to screen and treat HIV in people with TB, but no prevention strategies are mentioned (Table 5).

Table 6: According to policy documents and guidelines, what are clinicians asked to do regarding HIV?

Country	Preven t	Screen	Treat	Any
Brazil	70%	84%	79%	**97%**
Philippines	66%	96%	62%	**100%**
India	61%	64%	62%	**87%**
China	69%	64%	83%	**97%**
Nigeria	87%	94%	94%	**97%**
Uganda	89%	84%	89%	**95%**
Mozambique	91%	91%	91%	**91%**
Angola	17%	0%	17%	**17%**
Pakistan	43%	86%	71%	**100%**
Bangladesh	50%	67%	50%	**83%**
Kenya	80%	100%	80%	**100%**
Papua New Guinea	83%	100%	100%	**100%**

Note: Values ≥50% are shaded green and values <50% are shaded red. The column ‘Any’ summarises respondents that answered positively to any of the three sub-items (prevent, screen or treat).

DM – DM in people with TB is considered in policy documents and guidelines of Brazil, Philippines, China, Mozambique, Zimbabwe and Kenya, while it is not considered in Nigeria, Angola, nor the Democratic Republic of Congo. In the remaining countries, clinicians are asked to do at least one of the three actions, but responses were less consistent (Table 6).

Table 7: According to policy documents and guidelines, what are clinicians asked to do regarding DM?

Country	Preven t	Screen	Treat	Any
Brazil	56%	69%	67%	**86%**
Philippines	57%	98%	74%	**98%**
India	38%	51%	49%	**72%**
China	64%	61%	78%	**86%**
Nigeria	13%	19%	26%	**26%**
Uganda	42%	68%	58%	**79%**
Mozambique	55%	64%	73%	**82%**
Angola	0%	0%	0%	**0%**
Pakistan	29%	86%	57%	**86%**
Zimbabwe	67%	100%	100%	**100%**
Bangladesh	33%	67%	50%	**67%**
Kenya	60%	100%	60%	**100%**
Papua New Guinea	33%	33%	67%	**67%**
Sierra Leone	0%	80%	40%	**100%**
Democratic Republic of Congo	20%	20%	40%	**40%**

Note: Values ≥50% are shaded green and values <50% are shaded red. The column ‘Any’ summarises respondents that answered positively to any of the three sub-items (prevent, screen or treat)

Tobacco – While tobacco is considered in one way or another in most of the countries (with the notable exceptions of Nigeria, Angola, Papua New Guinea, and Democratic Republic of Congo), in many cases the focus of policy documents and guidelines seems to be on prevention, rather than screening and treatment, although there are discrepancies among respondents (Table 7).

Table 8: According to policy documents and guidelines, what are clinicians asked to do regarding tobacco?

Country	Prevent	Screen	Treat	Any
Brazil	54%	62%	53%	**81%**
Philippines	57%	72%	32%	**87%**
India	61%	46%	33%	**77%**
China	56%	44%	42%	**64%**
Nigeria	13%	10%	10%	**13%**
Uganda	58%	32%	21%	**68%**
Mozambique	55%	27%	0%	**55%**
Angola	33%	17%	17%	**33%**
Pakistan	14%	43%	14%	**57%**
Bangladesh	50%	17%	33%	**50%**
Zimbabwe	67%	67%	67%	**100%**
Kenya	100%	60%	40%	**100%**
Papua New Guinea	17%	17%	0%	**17%**
Zambia	50%	50%	50%	**50%**
Sierra Leone	40%	40%	0%	**60%**
Democratic Republic of Congo	40%	0%	0%	**40%**

Note: Values ≥50% are shaded green and values <50% are shaded red. The column 'Any' summarises respondents that answered positively to any of the three sub-items (prevent, screen or treat)

Alcohol – It is unclear to what extent alcohol use in people with TB is considered in policy documents or guidelines, as the proportion of respondents who answer positively to this question is not much higher than 50%. In Nigeria, Angola, Pakistan, Bangladesh, Papua New Guinea and the Democratic Republic of Congo this condition is clearly not considered. Notably, in none of the countries are clinicians asked to treat for alcohol use in people with TB, according to the policy documents and guidelines (Table 8).

Table 9: According to policy documents and guidelines, what are clinicians asked to do regarding alcohol?

Country	Prevent	Screen	Treat	Any
Brazil	51%	53%	44%	**75%**
Philippines	57%	62%	30%	**79%**
India	59%	43%	36%	**72%**
China	53%	39%	44%	**67%**
Nigeria	19%	16%	16%	**19%**
Uganda	63%	37%	26%	**74%**

Mozambique	55%	27%	0%	**55%**
Angola	17%	17%	17%	**17%**
Pakistan	0%	29%	14%	**29%**
Bangladesh	17%	0%	17%	**17%**
Kenya	100%	60%	40%	**100%**
Sierra Leone	40%	40%	0%	**60%**
Papua New Guinea	17%	17%	0%	**17%**
Democratic Republic of Congo	40%	0%	20%	**40%**

Note: Values ≥50% are shaded green and values <50% are shaded red. The column 'Any' summarises respondents that answered positively to any of the three sub-items (prevent, screen or treat)

Depression – Overall, depression is rarely considered in policy documents and guidelines. Only among respondents from Mozambique did the majority report that clinicians were asked to prevent, screen and treat for this condition in people with TB, although in the Philippines, India, China, Pakistan and Kenya there were (slightly) more than 50% of respondents who reported that at least one action (prevent, screen or treat) was required from clinicians (Table 9).

Table 10: According to policy documents and guidelines, what are clinicians asked to do regarding depression?

Country	Prevent	Screen	Treat	Any
Brazil	25%	31%	25%	**45%**
Philippines	43%	55%	42%	**68%**
India	38%	36%	41%	**57%**
China	50%	36%	56%	**67%**
Nigeria	10%	6%	6%	**10%**
Uganda	42%	37%	21%	**47%**
Mozambique	55%	82%	73%	**91%**
Angola	0%	0%	0%	**0%**
Pakistan	43%	43%	43%	**57%**
Bangladesh	17%	0%	17%	**17%**
Kenya	40%	60%	60%	**60%**
Sierra Leone	20%	20%	20%	**40%**
Papua New Guinea	17%	17%	17%	**17%**
Democratic Republic of Congo	0%	0%	40%	**40%**

Note: Values ≥50% are shaded green and values <50% are shaded red. The column 'Any' summarises respondents that answered positively to any of the three sub-items (prevent, screen or treat).

8b- What conditions are mentioned, and what are clinicians asked to do? - Overview

In Table 10 we present the results of countries with ≥10 responses for the 5 most common and concerning conditions, which gives a better overview of what comorbidities are considered in what countries. Results for all countries and all conditions can be found in the Supplement

According to the majority of respondents from **Brazil**, HIV, DM, and tobacco and alcohol use are considered in one way or another in the policy documents and guidelines, but not

depression. In **India**, clinicians are asked to prevent, screen, and treat HIV, according to the majority of respondents from this country, although there are discrepancies with regards to the other comorbidities. Based on the responses from the **Philippines**, HIV, DM, tobacco use and alcohol use in people with TB are addressed in the policy documents and guidelines, but not depression. In **China**, it appears that HIV and DM are addressed, but there are discrepancies among respondents when it comes to tobacco and alcohol use, and depression. Among respondents from **Nigeria** there appears to be an agreement in that HIV is considered in policy documents and guidelines, but not DM, tobacco and alcohol use, nor depression. In **Uganda**, there seems to be an agreement that HIV is considered in policy documents and guidelines and that depression is not, while responses are less consistent regarding DM and tobacco and alcohol use. Finally, responses from **Mozambique** stand out by the fact that depression does seem to be considered in the policy documents and guidelines, in addition to HIV and DM.

Table 11: According to policy documents and guidelines, what are clinicians asked to do regarding HIV, DM, tobacco and alcohol use, and depression? (Summary)

Country (responses)	Condition	Prevention	Screening	Treatment	Any
Brazil (102)	HIV	70%	84%	79%	**97%**
	DM	56%	69%	67%	**86%**
	TOB	54%	62%	53%	**81%**
	ALC	51%	53%	44%	**75%**
	DEP	25%	31%	25%	**45%**
India (61)	HIV	61%	64%	62%	**87%**
	DM	38%	51%	49%	**72%**
	TOB	61%	46%	33%	**77%**
	ALC	59%	43%	36%	**72%**
	DEP	38%	36%	41%	**57%**
Philippines (53)	HIV	66%	96%	62%	**100%**
	DM	57%	98%	74%	**98%**
	TOB	57%	72%	32%	**87%**
	ALC	57%	62%	30%	**79%**
	DEP	43%	55%	42%	**68%**
China (36)	HIV	69%	64%	83%	**97%**
	DM	64%	61%	78%	**86%**
	TOB	56%	44%	42%	**64%**
	ALC	53%	39%	44%	**67%**
	DEP	50%	36%	56%	**67%**
Nigeria (31)	HIV	87%	94%	94%	**97%**
	DM	13%	19%	26%	**26%**
	TOB	13%	10%	10%	**13%**
	ALC	19%	16%	16%	**19%**
	DEP	10%	6%	6%	**10%**
Uganda (19)	HIV	89%	84%	89%	**95%**
	DM	42%	68%	58%	**79%**
	TOB	58%	32%	21%	**68%**

	ALC	63%	37%	26%	**74%**
	DEP	42%	37%	21%	**47%**
Mozambique (11)	HIV	91%	91%	91%	**91%**
	DM	55%	64%	73%	**82%**
	TOB	55%	27%	0%	**55%**
	ALC	55%	27%	0%	**55%**
	DEP	55%	82%	73%	**91%**

Note: Values ≥50% are shaded green and values <50% are shaded red. The column 'Any' summarises respondents that answered positively to any of the three (prevent, screen or treat). Only countries with ≥10 responses and only the five most common and concerning conditions are presented (see Supplement for all countries and all conditions). DM: Diabetes mellitus; TOB: Tobacco use; ALC: Alcohol use; DEP: Depression

TB Multimorbidity in practice – Screening, diagnosis, surveillance and treatment...

In this section of the survey, we were interested in collecting data about how TB Multimorbidity was taken into account in practice, in the TB services. As in the previous section we considered in our analyses, in addition to the prespecified conditions, the ones most often referred to in the open-text fields: Covid-19, drug use, hypertension and malnutrition. Also like in the previous section, in this report we will focus on HIV, DM, tobacco use, alcohol use, and depression, but results for all comorbidities can be found in the Supplement.

9- Do you work in a TB clinic?

About half of the respondents worked in a TB clinic (Figure 7).

Figure 10: Do you work in a TB clinic?

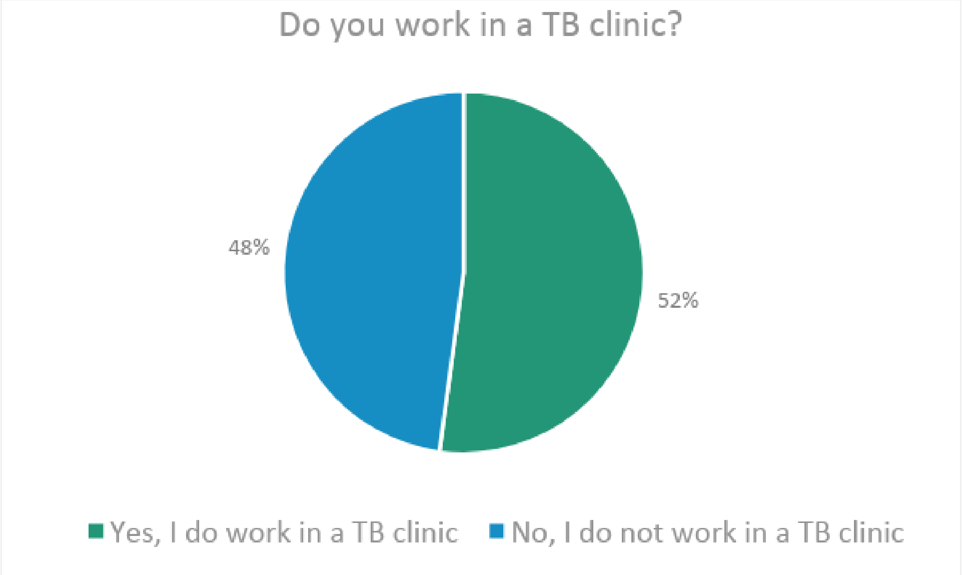

10- Regarding screening, diagnosis and surveillance...

This item consisted of five sub-items about what conditions are (1) diagnosed and (2) screened for in the respondent's TB service, and for what conditions the respondent's TB service (3) routinely collects data, (4) reports to the TB programme, or (5) reports to other disease control programmes.

In most of the countries with ≥10 responses (Table 11), the majority of respondents reported that **HIV** was diagnosed, screened for, data was collected, and cases were reported to the

TB programme or other programmes. Only among respondents from India were the responses heterogeneous. **DM** was considered (mainly diagnosed and screened for) in the majority of respondents' TB services in the Philippines and China and only in a minority (or not at all) of services in Nigeria and Mozambique. Both **Tobacco** and **Alcohol** use were diagnosed and screened for in some of the respondents' TB services in most of the countries. Only in Nigeria was this clearly not done. **Depression** was generally not considered in the respondents' TB services.

Considering the number of countries with ≥50% of respondents who answered positively (Table 12) we can see that over 20 of 27 countries had a majority of respondents reporting that **HIV** is considered in their TB service (diagnosis, screening, data collection, reporting to TB programme or reporting to other programmes). For **DM**, only 13 of 27 countries had a majority of its respondents reporting that this condition was screened for in their TB service. The number of countries with a majority of positive responses is 6 and 5 out of 27 for **tobacco** and **alcohol** use, respectively, and for **depression** this number is only 3 countries out of 27.

Table 12: Percentage of respondents whose TB services perform tasks for screening, diagnosis and surveillance for the most common and concerning conditions

Country (responses)	Condition	Diagnose	Screen	Collect data	Report to TBP	Report to other programmes
Brazil (92)	HIV	85%	78%	84%	77%	72%
	DM	65%	54%	54%	49%	34%
	TOB	61%	51%	45%	46%	30%
	ALC	59%	52%	47%	47%	32%
	DEP	38%	21%	15%	13%	26%
India (61)	HIV	61%	52%	43%	44%	44%
	DM	49%	44%	43%	43%	38%
	TOB	49%	34%	30%	31%	20%
	ALC	44%	28%	26%	33%	18%
	DEP	26%	20%	20%	21%	15%
Philippines (50)	HIV	72%	80%	68%	76%	62%
	DM	74%	76%	60%	44%	38%
	TOB	46%	48%	30%	26%	18%
	ALC	40%	44%	26%	24%	16%
	DEP	32%	32%	16%	12%	10%
China (36)	HIV	83%	72%	72%	67%	72%
	DM	83%	75%	75%	53%	47%
	TOB	47%	42%	50%	22%	17%
	ALC	47%	42%	50%	25%	17%
	DEP	42%	36%	39%	25%	19%
Nigeria (30)	HIV	93%	97%	93%	90%	87%
	DM	13%	0%	0%	0%	0%
	TOB	3%	10%	0%	0%	0%
	ALC	7%	10%	0%	0%	0%
	DEP	7%	3%	0%	0%	0%

Uganda (19)	HIV	84%	79%	74%	74%	58%
	DM	63%	47%	21%	21%	26%
	TOB	47%	42%	37%	21%	21%
	ALC	42%	42%	32%	16%	16%
	DEP	21%	21%	11%	16%	11%
Mozambique (11)	HIV	91%	91%	91%	91%	100%
	DM	27%	18%	27%	18%	20%
	TOB	45%	45%	18%	18%	20%
	ALC	55%	36%	18%	18%	20%
	DEP	45%	45%	18%	9%	20%

Note: Values ≥66% are shaded green, values ≤ 33% are shaded red, values between 33% and 66% are shaded orange. Only countries with ≥10 responses and only the five most common and concerning conditions are presented (see Supplement for all countries and all conditions). DM: Diabetes mellitus; TOB: Tobacco use; ALC: Alcohol use; DEP: Depression

Table 13: Number of countries (with at least one response) where ≥50% of respondents answered positively to each sub-item of item 10

Condition	Diagnose	Screen	Collect data	Report to TBP	Report to other programmes	Number of countries
HIV	24	21	21	20	20	*27*
DM	13	7	6	5	5	*27*
TOB	6	5	1	1	3	*27*
ALC	5	4	1	1	3	*27*
DEP	3	1	0	1	3	*27*

DM: Diabetes mellitus; TOB: Tobacco use; ALC: Alcohol use; DEP: Depression

11- Regarding treatment...

This item consisted of seven sub-items about for what conditions is the respondent's TB service (1) able to start care, (2) offer maintenance care, (3) refer patients to a specialised service, (4) liaise with other services, (5) provide medication, (6) train the service providers, and (7) what conditions are covered in supervision, monitoring and evaluation.

Among the countries with ≥10 responses (Table 13; results for all conditions and countries can be found in the Supplement), treatment for **HIV** was started and/or maintained in the majority of the respondents' TB services in Nigeria, Uganda, and Mozambique, while in Brazil, the Philippines, and China, most of the respondents' TB services referred to or liaised with other specialised services. **DM** was treated in the majority of the respondents' TB services in the Philippines and China (with those in the latter also mostly providing medication), while only a minority of respondents from Nigeria, Uganda and Mozambique reported any actions related to care. **Tobacco** and **Alcohol** use, and **Depression** were considered for treatment of management only in a minority of Tb services in all countries.

Considering the number of countries (with at least one response) with ≥50% of respondents who answered positively to each item (Table 14; results for all conditions can be found in the Supplement) we can see that 19 of 27 countries had a majority of respondents reporting that care for **HIV** was started in their TB service. For **DM**, only 7 of 27 countries had a majority of its respondents reporting that care for this condition was started in their TB service. Treatment for **tobacco** and **alcohol** use and for **depression** was rarely considered.

Table 14: Percentage of respondents whose TB services perform tasks for screening, diagnosis and surveillance for the most common and concerning conditions

Country (responses)	Condition	Start care	Maintenance of care	Referral to specialised services	Liaison with other services	Provide medication	Training of service providers	Supervision, monitoring and evaluation
Brazil (92)	HIV	55%	52%	72%	70%	48%	58%	61%
	DM	55%	47%	34%	28%	39%	24%	38%
	TOB	42%	25%	41%	33%	23%	22%	33%
	ALC	34%	20%	47%	30%	10%	12%	30%
	DEP	26%	24%	48%	28%	24%	10%	18%
India (61)	HIV	49%	44%	38%	34%	38%	33%	41%
	DM	38%	36%	26%	30%	31%	23%	30%
	TOB	28%	15%	26%	20%	16%	18%	23%
	ALC	25%	18%	25%	20%	15%	18%	20%
	DEP	21%	15%	25%	15%	16%	18%	16%
Philippines (50)	HIV	60%	36%	74%	66%	30%	74%	64%
	DM	72%	64%	44%	36%	52%	32%	52%
	TOB	20%	10%	26%	16%	8%	12%	12%
	ALC	20%	10%	20%	16%	8%	8%	12%
	DEP	24%	14%	48%	38%	10%	8%	16%
China (36)	HIV	58%	58%	75%	64%	53%	53%	58%
	DM	78%	78%	50%	44%	72%	64%	61%
	TOB	47%	50%	25%	28%	36%	44%	44%
	ALC	50%	44%	28%	31%	33%	42%	42%
	DEP	44%	39%	44%	50%	31%	31%	44%
Nigeria (30)	HIV	80%	70%	60%	80%	57%	83%	87%
	DM	3%	3%	23%	13%	7%	3%	7%
	TOB	0%	3%	0%	3%	0%	3%	0%
	ALC	0%	3%	0%	3%	0%	3%	0%
	DEP	0%	3%	13%	13%	0%	0%	0%
Uganda (19)	HIV	68%	72%	37%	47%	68%	74%	68%
	DM	26%	22%	42%	11%	11%	21%	26%
	TOB	16%	17%	11%	26%	5%	11%	21%
	ALC	5%	11%	16%	16%	0%	5%	21%
	DEP	26%	22%	37%	11%	11%	0%	5%
Mozambique (11)	HIV	82%	70%	27%	82%	91%	91%	91%
	DM	18%	20%	55%	45%	9%	27%	36%
	TOB	18%	10%	27%	36%	18%	27%	36%
	ALC	18%	10%	45%	36%	18%	27%	27%
	DEP	18%	20%	64%	55%	9%	55%	55%

Note: Values ≥66% are shaded green, values ≤ 33% are shaded red, values between 33% and 66% are shaded orange. Only countries with ≥10 responses and only the five most common and concerning conditions are presented (see Supplement for all countries and all conditions). DM: Diabetes mellitus; TOB: Tobacco use; ALC: Alcohol use; DEP: Depression

Table 15: Number of countries (with at least one response) where ≥50% of respondents answered positively to each sub-item of item 11

Condition	Start care	Maintenance of care	Referral to specialised services	Liaison with other services	Provide medication	Training of service providers	Supervision, monitoring and evaluation	Number of countries
HIV	19	14	15	16	13	19	19	*27*
DM	7	6	8	7	4	7	8	*27*
TOB	2	2	2	2	0	4	4	*27*
ALC	1	1	2	2	0	3	3	*27*
DEP	2	2	3	3	0	3	2	*27*

DM: Diabetes mellitus; TOB: Tobacco use; ALC: Alcohol use; DEP: Depression

10+11 Overview of screening and treatment

To have a better overview of the responses, we decided to combine several responses into new variables as follows: 'Diagnose / Screen' would be positive if either diagnosis or screening in item 10 were answered positively; 'Start / Maintain care' would be positive if the respondents' TB service either started or did maintenance care (item 11); and when TB services referred to specialised services or liaised with other services (also item 11) we would register the response in the new variable 'Referral / Liaison' as positive.

In all the countries with ≥10 responses (results for all countries and conditions can be found in the Supplement), the majority of TB services diagnose or screen for **HIV** and either start or maintain care, or refer patients to or liaise with specialised services. **DM** is screened for or diagnosed in the majority of the respondents' TB services in Brazil, the Philippines, China and Uganda, but of these countries, only in the Philippines and China was there a majority of TB services that started or maintained care. **Tobacco** and **Alcohol** use was screened for or diagnosed in a majority of the respondents' TB services only in Brazil, although only about half of the respondents indicated that care was started or maintained or there was a referral to or liaison with specialised services. **Depression** was not consistently screened for or diagnosed in any of the countries (Table 15).

Considering the number of countries (with at least one response) where ≥50% of responses in the combined variables of items 10 and 11 were positive, **HIV** was considered in most of the countries (most of the TB services of 24 of 27 countries screened for or diagnosed HIV, and in 20 of 27 countries treatment was started or maintained in the majority of TB services). In half of the countries (14 of 27) was **DM** consistently screened for or diagnosed, with a similar number of countries (13 of 27) where most TB services referred to or liaised with specialised services. Regarding **Tobacco** use, most of the TB services in 13 of 27 countries screened for or diagnosed tobacco use, although in few countries did TB services consistently start or maintain care or refer to or liaise with specialised services. **Alcohol** use and **depression** were consistently considered in only a small number of countries (Table 16). The full list of conditions can be found in the Supplement.

Table 16: Percentage of respondents whose TB services screen for or diagnose each of the five most common and concerning conditions, start or maintain care, and refer or liaise with specialised services (only countries with ≥10 responses shown).

Country (responses)	Condition	Diagnose / Screen	Start / Maintain care	Referral / Liaison

Brazil (92)	HIV	87%	59%	80%
	DM	71%	59%	45%
	TOB	66%	45%	51%
	ALC	68%	37%	52%
	DEP	43%	32%	52%
India (61)	HIV	62%	54%	43%
	DM	52%	43%	33%
	TOB	52%	30%	28%
	ALC	49%	26%	28%
	DEP	31%	26%	26%
Philippines (50)	HIV	86%	62%	84%
	DM	80%	72%	58%
	TOB	58%	22%	30%
	ALC	52%	22%	28%
	DEP	40%	26%	60%
China (36)	HIV	86%	64%	78%
	DM	86%	83%	56%
	TOB	53%	53%	31%
	ALC	50%	56%	33%
	DEP	44%	44%	61%
Nigeria (30)	HIV	97%	83%	83%
	DM	13%	3%	27%
	TOB	13%	3%	3%
	ALC	13%	3%	3%
	DEP	10%	3%	20%
Uganda (19)	HIV	84%	74%	53%
	DM	68%	32%	47%
	TOB	53%	26%	32%
	ALC	42%	11%	26%
	DEP	26%	32%	47%
Mozambique (11)	HIV	91%	82%	82%
	DM	36%	18%	73%
	TOB	55%	18%	45%
	ALC	55%	18%	64%
	DEP	55%	18%	73%

Note: Values ≥66% are shaded green, values ≤ 33% are shaded red, values between 33% and 66% are shaded orange. Only countries with ≥10 responses and only the five most common and concerning conditions are presented (see Supplement for all countries and all conditions). DM: Diabetes mellitus; TOB: Tobacco use; ALC: Alcohol use; DEP: Depression

Table 17: Number of countries (with at least one response) where ≥50% of responses in the combined variables of items 10 and 11 were positive

Condition	Diagnose / Screen	Start / Maintain care	Referral / Liaison	Number of countries
HIV	24	20	19	*27*

DM	14	7	13	*27*
TOB	13	3	4	*27*
ALC	8	2	5	*27*
DEP	5	2	8	*27*

DM: Diabetes mellitus; TOB: Tobacco use; ALC: Alcohol use; DEP: Depression

11b- Is medication provided for free?

Whenever a respondent answered that medication for any condition was provided by their TB service, we were also interested in knowing if it was provided for free. As shown in Table 17, in almost all TB services where medication was provided for **HIV**, it was done for free. For **DM** and **Depression**, when medication was provided it was mostly done for free in TB services in some countries, but not in others. There were few respondents who reported that medication to treat **tobacco** and **alcohol** use was provided by their TB service. The majority of those respondents from Brazil, India and Mozambique reported that these medications were provided for free, while this was not the case for respondents from China, Kenya and the Philippines. The full list including all conditions can be found in the Supplement.

Table 18: When medication is provided by the TB service, is it provided for free?

Country	HIV	DM	Tobacco use	Alcohol use	Depression
Brazil	100%	100%	94%	88%	100%
India	100%	88%	100%	100%	100%
Philippines	100%	70%	50%	0%	60%
China	68%	12%	31%	17%	0%
Nigeria	100%	0%	.	.	.
Uganda	100%	100%	.	.	100%
Mozambique	100%	100%	100%	100%	100%
Angola	.	.	.	.	.
Pakistan	100%	0%	.	.	0%
Sierra Leone	100%	.	.	.	.
Bangladesh	100%	0%	.	.	0%
Kenya	75%	0%	0%	0%	0%
Papua New Guinea	100%	.	.	.	0%
Zimbabwe	100%	.	.	.	.
Democratic Republic of Congo	.	.	.	.	.
Zambia	100%	100%	.	.	.
South Africa	100%	100%	.	.	100%
Lesotho	100%	.	.	.	.
United Republic of Tanzania	100%	.	.	.	.
Congo (Brazzaville)	100%	.	.	.	.
Ethiopia	.	.	.	.	.
Myanmar	.	100%	.	.	.
Russian Federation	.	.	.	.	.
Indonesia	.	.	.	.	.
Liberia	100%	.	.	.	.
Central African Republic	.	.	.	.	.
Namibia	100%	.	.	.	.

Note: Percentages represent affirmative responses. DM: Diabetes mellitus.

Respondents' experience working in a TB service / clinic

In this section of the survey, we were interested in the respondents' experience working in a TB service: what the most common conditions were in their practice and which conditions they were most concerned about, as well as how capable they felt to diagnose and/or treat each condition.

13- In your setting, and in your experience, what are the three most common conditions you find in people with TB?

When we pooled the three most common conditions reported by all the respondents, HIV, DM, Tobacco and Alcohol use, and Depression were the 5 most common conditions (Figure 8 A). However, given the large differences between countries in the number of responses, we decided to use a different approach: First, we identified the 3 most common conditions in each country (see Supplement for individual country's list of conditions), and then we pooled together the 3 most common conditions in each country (rather than from each respondent). This approach yielded the same list of 5 most common conditions: HIV, DM, Tobacco and Alcohol use, and Depression (Figure 8 B).

Figure 11: The most common conditions in people with TB, according to respondents

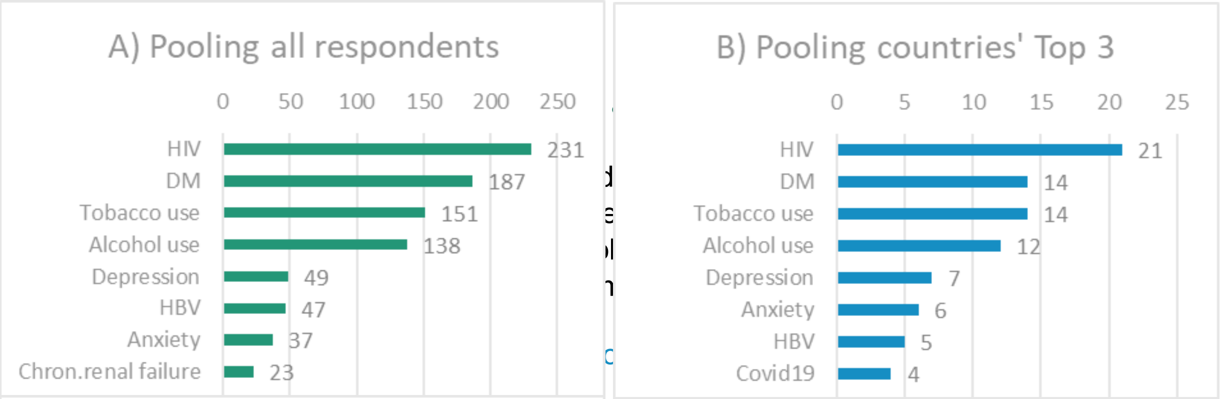

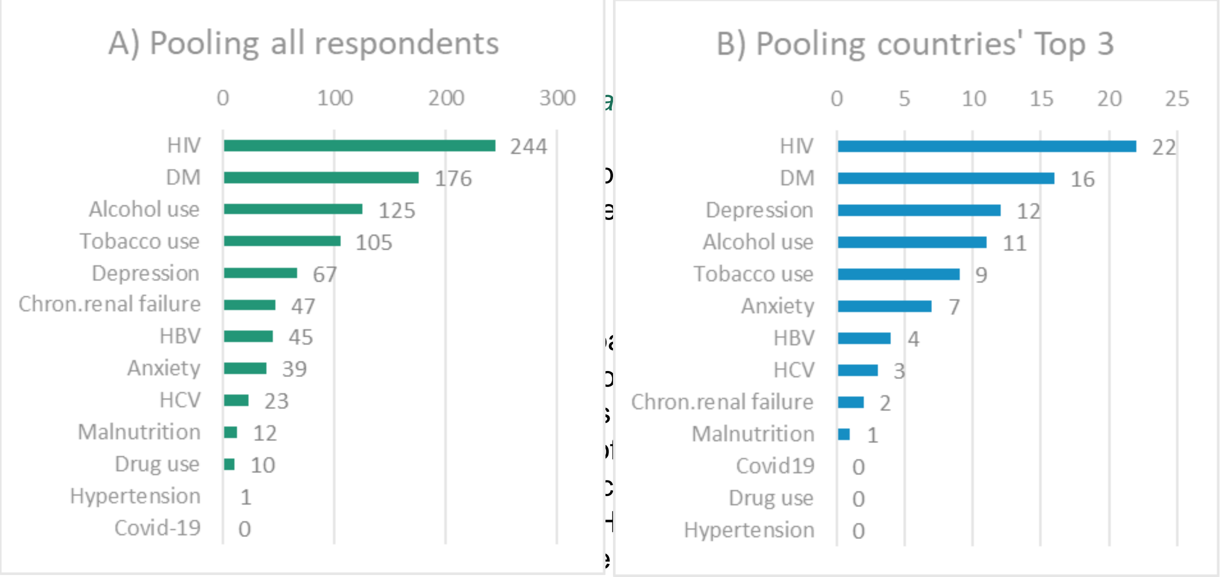

diagnose and/or treat them.

Considering the number of countries (with at least one response) with ≥50% of respondents who answered positively (Table 19) we can see that in 15 and 14 of 19 countries the majority of respondents felt capable of diagnosing **HIV** and **DM**, respectively, in people with TB. In 12 and 13 countries, respectively, did most of the respondents feel capable of treating these

conditions as well. In half of the countries did the majority of respondents feel capable of diagnosing **Tobacco** or **Alcohol** use, although in only three countries did the majority of respondents feel capable of treating these conditions. Regarding **Depression**, in only 5 countries out of 19 did ≥50% of respondents feel capable of diagnosing this condition, and only in one country did the majority feel capable of treating it as well.

Table 19: How capable do the respondents feel regarding diagnosing and/or treating each condition

Country (responses)	Condition	Capable of diagnosing	Capable of treating
Brazil (47)	HIV	94%	40%
	DM	79%	53%
	TOB	81%	45%
	ALC	74%	15%
	DEP	49%	13%
China (31)	HIV	84%	45%
	DM	94%	68%
	TOB	65%	42%
	ALC	68%	52%
	DEP	48%	23%
Philippines (29)	HIV	93%	10%
	DM	93%	79%
	TOB	62%	34%
	ALC	55%	21%
	DEP	62%	31%
India (26)	HIV	81%	62%
	DM	54%	54%
	TOB	46%	46%
	ALC	46%	46%
	DEP	50%	46%

Note: Values ≥66% are shaded green, values ≤ 33% are shaded red, values between 33% and 66% are shaded orange. Only countries with ≥10 responses and only the five most common and concerning conditions are presented (see Supplement for all countries and all conditions). DM: Diabetes mellitus; TOB: Tobacco use; ALC: Alcohol use; DEP: Depression

Table 20: Number of countries (with at least one response) where ≥50% of respondents answered positively to each sub-item of item 15

Condition	Capable of diagnosing	Capable of treating	Number of countries
HIV	15	12	*19*
DM	14	13	*19*
TOB	9	3	*19*
ALC	9	3	*19*
DEP	5	1	*19*

DM: Diabetes mellitus; TOB: Tobacco use; ALC: Alcohol use; DEP: Depression

Overview: TB Multimorbidity in theory, in practice, and perceived capability...

In order to have a better overview of the results, we wanted to consider how each comorbidity is considered in theory (what are clinicians asked to do in the policy documents and guidelines), in practice (diagnose/screening, start/maintain care, and referral to/liaison with specialised services), and how capable respondents feel to diagnose and treat them.

Table 20 summarises the number of countries with at least one response in all the summarised items (8b, 10, 11, and 15), which limits the total number of countries to 19. Therefore, the exact numbers might differ from when individual items were summarised further up. When comparing the considerations of interventions in theory (policy documents and guidelines) and in practice, it is notable that there are more countries where the majority of respondents diagnose or screen for many of the conditions, than countries where these conditions are considered in theory.

HIV is considered in the policy documents and guidelines in 14 of 19 countries, and in 14 or more countries the majority of respondents indicated that they took this condition into account in some way in practice. **DM** is considered in theory only in 6 of 19 countries, but in 10 countries the majority of respondents screened for and/or diagnosed this condition. Furthermore, in 14 of 19 countries the majority of respondents felt capable of screening for this condition, and in 13 countries they felt capable of treating it. Regarding **Tobacco** use, in about half of the countries was this condition considered in theory and screened for and/or diagnosed in practice by the majority of respondents. In a similar number of countries, the majority of respondents felt capable of diagnosing this condition, but only in 3 countries did most of the respondents feel capable of treating it. **Alcohol** use was considered in policy documents and guidelines of 8 of 19 countries, but only in 6 countries did most of the respondents screen for it. Finally, **Depression** was considered only in 2 of 19 countries, although in 6 countries there was a majority of respondents who referred patients to or liaised with specialised services for this condition.

Table 21: Number of countries (with at least one response) where ≥50% of respondents answered positively to each sub-item of items 8b, 10 (combined), 11 (combined), and 15

Condition	Theory	Practice			Capable		Number of countries
	Prevention / Screening / Treatment	Diagnose / Screen	Start / Maintain care	Referral / Liaison	Diagnose	Treat	
HIV	14	17	14	14	15	12	*19*
DM	6	10	6	9	14	13	*19*
TOB	9	10	3	3	9	3	*19*
ALC	8	6	2	4	9	3	*19*
DEP	2	3	1	6	5	1	*19*

DM: Diabetes mellitus; TOB: Tobacco use; ALC: Alcohol use; DEP: Depression

Discussion

In this cross-sectional survey we aimed to have a better understanding of the extent to which TB multimorbidity is recognised and addressed in policy, management and services within TB healthcare, which specific comorbidities are being considered in TB healthcare, and to identify potential gaps in service provision for TB multimorbidity in high-burden countries.

We identified the five conditions that were most common and concerning, according to the respondents' experience: HIV, DM, tobacco use, alcohol use, and depression. We found that

most of respondents of almost all countries (all except Angola) indicated that, according to policy documents and guidelines, clinicians should prevent, screen for, and treat HIV in people with TB. DM was considered in less countries, and there were more discrepancies between respondents. Regarding tobacco and alcohol use, most of the respondents of most of the countries indicated that clinicians were not asked to screen or treat for these conditions, and there were discrepancies regarding prevention. Only a minority of respondents of most countries (except in Mozambique) said that depression was considered in some way in policy documents and guidelines.

At the level of service provision, HIV in people with TB was considered in most of the countries (most of the TB services of 24 of 27 countries screened for or diagnosed HIV, and in 20 of 27 countries treatment was started or maintained in the majority of TB services), and in about half of the countries with at least one response DM was consistently screened for, diagnosed, patients were referred to specialised services, or the TB services liaised with specialised services for DM. Regarding tobacco use, most of the TB services in 6and 5 of 27 countries screened for or diagnosed tobacco and alcohol use, respectively, and in fewer countries did TB services consistently start or maintain care or refer to or liaise with specialised services. Depression was consistently considered in only a small number of countries.

There does not appear to be any obvious discrepancies between the most concerning conditions for respondents and their considerations in policy and practice.

Strengths and limitations

Our sampling strategy resulted in over 400 responses from 27 of 33 high-burden countries (30 from the current list + three that were recently removed from it), although two thirds of all respondents come from only four countries (Brazil, India, Philippines and China). Our results are therefore not representative of high-burden countries overall, nor are the responses from each individual country necessarily representative of that country’s policy. However, we got responses from TB program managers, primary, secondary and tertiary health care workers, and consultants. We therefore believe that our results do provide valuable initial insight into this topic, particularly for the countries with multiple respondents.

Conclusion

Despite the limitations resulting from a non-representative sampling procedure, this survey provides some insight into how TB multimorbidity is considered both in theory and in practice in high-TB-burden countries.

References

1. World Health Organisation. Tuberculosis (TB) [Internet]. [cited 2020 May 21]. Available from: https://www.who.int/news-room/fact-sheets/detail/tuberculosis

2. Foguet-Boreu Q, Violan C, Roso-Llorach A, Rodriguez-Blanco T, Pons-Vigués M, Muñoz-Pérez MA, et al. Impact of multimorbidity: acute morbidity, area of residency and use of health services across the life span in a region of south Europe. BMC Fam Pract. 2014 Mar 26;15(1):55.

3. Duko B, Gebeyehu A, Ayano G. Prevalence and correlates of depression and anxiety among patients with tuberculosis at WolaitaSodo University Hospital and Sodo Health Center, WolaitaSodo, South Ethiopia, Cross sectional study. BMC Psychiatry. 2015 Sep 14;15(1):214.

4. Gelaw YA, Williams G, Soares Magalhaes RJ, Gilks CF, Assefa Y. HIV Prevalence Among Tuberculosis Patients in Sub-Saharan Africa: A Systematic Review and Meta-analysis. AIDS Behav. 2019;23(6):1561–75.

5. Noubiap JJ, Nansseu JR, Nyaga UF, Nkeck JR, Endomba FT, Kaze AD, et al. Global prevalence of diabetes in active tuberculosis: a systematic review and meta-analysis of data from 2.3 million patients with tuberculosis. Lancet Glob Health. 2019;7(4):e448–60.

6. Soares LN, Spagnolo LM de L, Tomberg JO, Zanatti CL de M, Cardozo-Gonzales RI. Relationship between multimorbidity and the outcome of the treatment for pulmonary tuberculosis. Rev Gaúcha Enferm. 2020;41.

7. Ruiz-Grosso P, Cachay R, de la Flor A, Schwalb A, Ugarte-Gil C. Association between tuberculosis and depression on negative outcomes of tuberculosis treatment: A systematic review and meta-analysis. PloS One. 2020;15(1):e0227472.

8. Huddart S, Svadzian A, Nafade V, Satyanarayana S, Pai M. Tuberculosis case fatality in India: a systematic review and meta-analysis. BMJ Glob Health. 2020;5(1):e002080.

9. Eshetie S, Gizachew M, Alebel A, van Soolingen D. Tuberculosis treatment outcomes in Ethiopia from 2003 to 2016, and impact of HIV co-infection and prior drug exposure: A systematic review and meta-analysis. PloS One. 2018;13(3):e0194675.

10. Gautam S, Shrestha N, Mahato S, Nguyen TPA, Mishra SR, Berg-Beckhoff G. Diabetes among tuberculosis patients and its impact on tuberculosis treatment in South Asia: a systematic review and meta-analysis. Sci Rep. 2021 Jan 22;11(1):2113.

11. Sculier D, H̲āylayasus Gétāhun, World Health Organization. WHO policy on collaborative TB/HIV activities: guidelines for national programmes and other stakeholders [Internet]. 2012 [cited 2021 Jun 8]. Available from: http://whqlibdoc.who.int/publications/2012/9789241503006_eng_Annexes.pdf

12. Strategic and Technical Advisory Group for TB (STAG-TB). Use of high burden country lists for TB by WHO in the post-2015 era. [Internet]. World Health Organization (WHO); 2015 [cited 2021 Jun 12]. Available from: https://www.who.int/tb/publications/global_report/high_tb_burdencountrylists2016-2020.pdf

13. World Health Organization. WHO global lists of high burden countries for TB, multidrug/rifampicin-resistant TB (MDR/RR-TB) and TB/HIV, 2021–2025 [Internet]. Geneva: World Health Organization; 2021 [cited 2021 Jun 18]. Available from: https://www.who.int/news/item/17-06-2021-who-releases-new-global-lists-of-high-burden-countries-for-tb-hiv-associated-tb-and-drug-resistant-tb
